# UBA1/GARS-dependent pathways drive sensory-motor connectivity defects in spinal muscular atrophy

**DOI:** 10.1093/brain/awy237

**Published:** 2018-09-25

**Authors:** Hannah K Shorrock, Dinja van der Hoorn, Penelope J Boyd, Maica Llavero Hurtado, Douglas J Lamont, Brunhilde Wirth, James N Sleigh, Giampietro Schiavo, Thomas M Wishart, Ewout J N Groen, Thomas H Gillingwater

**Affiliations:** 1 Euan MacDonald Centre for Motor Neurone Disease Research, University of Edinburgh, Edinburgh, UK; 2 Edinburgh Medical School: Biomedical Sciences, University of Edinburgh, Edinburgh, UK; 3 Roslin Institute, Royal (Dick) School of Veterinary Science, University of Edinburgh, UK; 4 FingerPrints Proteomics Facility, University of Dundee, UK; 5 Institute of Human Genetics, Center for Molecular Medicine Cologne, Institute for Genetics and Center for Rare Diseases Cologne, University of Cologne, Germany; 6 Sobell Department of Motor Neuroscience and Movement Disorders, Institute of Neurology, University College London, UK; 7 Discoveries Centre for Regenerative and Precision Medicine, University College London Campus, London, UK; 8 UK Dementia Research Institute at UCL, London, UK

**Keywords:** motor neuron disease, spinal muscular atrophy, Charcot-Marie-Tooth disease, UBA1, GARS

## Abstract

Deafferentation of motor neurons as a result of defective sensory-motor connectivity is a critical early event in the pathogenesis of spinal muscular atrophy, but the underlying molecular pathways remain unknown. We show that restoration of ubiquitin-like modifier-activating enzyme 1 (UBA1) was sufficient to correct sensory-motor connectivity in the spinal cord of mice with spinal muscular atrophy. Aminoacyl-tRNA synthetases, including GARS, were identified as downstream targets of UBA1. Regulation of GARS by UBA1 occurred via a non-canonical pathway independent of ubiquitylation. Dysregulation of UBA1/GARS pathways in spinal muscular atrophy mice disrupted sensory neuron fate, phenocopying GARS-dependent defects associated with Charcot-Marie-Tooth disease. Sensory neuron fate was corrected following restoration of UBA1 expression and UBA1/GARS pathways in spinal muscular atrophy mice. We conclude that defective sensory motor connectivity in spinal muscular atrophy results from perturbations in a UBA1/GARS pathway that modulates sensory neuron fate, thereby highlighting significant molecular and phenotypic overlap between spinal muscular atrophy and Charcot-Marie-Tooth disease.

## Teaser

Deafferentation of motor neurons as a result of defective sensory-motor connectivity in the spinal cord is an important early event in spinal muscular atrophy. Shorrock *et al*. report that this process is reversible in mice and results from disruption of a novel UBA1/GARS pathway that controls sensory neuron fate.

## Introduction

Spinal muscular atrophy (SMA) is an autosomal recessive form of motor neuron disease with an incidence of 1 in ∼10 000 live births (Sugarman *et al.*, 2012; Verhaart *et al.*, 2017; Groen *et al.*, 2018*b*). SMA is characterized primarily by degeneration of lower motor neurons in the anterior horn of the spinal cord. This is accompanied by proximal muscle weakness and atrophy, resulting in a progressive decline in motor function and, in severe cases, paralysis and death (Kolb and Kissel, 2011; Shorrock *et al.*, 2018). Recently, compelling evidence has been put forward from several studies demonstrating that sensory neuron defects are a significant feature of SMA pathogenesis. For example, a reduction in numbers of myelinated dorsal root axons has been reported (Ling *et al.*, 2010), alongside significant reductions in the number of proprioceptive synapses formed onto motor neurons in the spinal cord (Ling *et al.*, 2010; Mentis *et al.*, 2011; Fletcher *et al.*, 2017). The deafferentation of motor neurons as a result of defective sensory-motor connectivity is now known to be a critical early event in the pathogenesis of SMA, where it represents a primary cause of motor neuron dysfunction (Ling *et al.*, 2010; Mentis *et al.*, 2011; Simon *et al.*, 2017). Similarly, it has previously been shown that sensory nerve endings innervating embryonic footpads display defective outgrowth and altered neuron terminal structure in SMA mice (Jablonka *et al.*, 2006). Although defects in sensory neuron outgrowth and altered β-actin protein and mRNA localization have been reported in cultured sensory neurons from SMA mice (Jablonka *et al.*, 2006), the molecular mechanisms underlying defective sensory-motor connectivity in SMA are still to be established.

In ∼95% of cases, SMA is caused by homozygous deletion of the survival motor neuron 1 gene (*SMN1*), which encodes the survival motor neuron protein (SMN) (Lefebvre *et al.*, 1995). Interestingly, however, not all forms of SMA are caused by deletions or mutations in the *SMN1* gene. Mutations in the ubiquitin-like modifier activating enzyme 1 gene (*UBA1*) cause a rare form of SMA known as X-linked SMA (XL-SMA) (Ramser *et al.*, 2008; Dlamini *et al.*, 2013; Jedrzejowska *et al.*, 2015). *UBA1* encodes the primary enzyme, UBA1, responsible for activating ubiquitin as the first step in the ubiquitin-conjugation pathway, to mark proteins for degradation by the proteasome (Groen and Gillingwater, 2015). XL-SMA is clinically similar to SMA and is characterized by loss of lower motor neurons, muscle weakness, hypotonia and a lack of reflexes. In addition, congenital contractures and fractures are also commonly associated with XL-SMA (Ramser *et al.*, 2008; Dlamini *et al.*, 2013; Jedrzejowska *et al.*, 2015). Mutations in *UBA1* underlying XL-SMA are predicted to cause instability of the enzyme, leading to a reduction in expression levels of UBA1 (Ramser *et al.*, 2008; Lao *et al.*, 2012; Balak *et al.*, 2017).

Recent findings have suggested that altered ubiquitin homeostasis is a core molecular feature of *SMN1*-dependent SMA, with reduced UBA1 expression central to this disruption in a range of animal models and in SMA patient-derived iPSC motor neurons (Aghamaleky Sarvestany *et al.*, 2014; Wishart *et al.*, 2014; Fuller *et al.*, 2015; Powis *et al.*, 2016). Suppression or pharmacological inhibition of UBA1 is sufficient to induce an SMA-like neuromuscular phenotype in zebrafish (Wishart *et al.*, 2014) and, similarly, pharmacological inhibition of UBA1 in Schwann cells recapitulates the defective myelination phenotype observed in SMA (Aghamaleky Sarvestany *et al.*, 2014; Hunter *et al.*, 2014). Consistent with this, therapies that restore UBA1 have shown beneficial effects on neuromuscular phenotypes in several SMA models (Powis *et al.*, 2016). SMA mice treated with adeno-associated virus serotype 9 (AAV9) expressing full-length human *UBA1* cDNA (AAV9-UBA1) show increased survival compared to untreated SMA mice, along with improvements in systemic (e.g. heart and liver) pathology. SMA mice treated with AAV9-UBA1 also showed a rescue of the number of motor neuron cell bodies in the spinal cord, an increase in muscle fibre diameter, and rescue of NMJ innervation defects compared to untreated SMA mice (Powis *et al.*, 2016).

Because of the wide ranging therapeutic benefits of treatment with AAV9-UBA1 and the relevance of UBA1-mediated degeneration for multiple types of SMA, here, we set out to determine whether UBA1-dependent pathways are responsible for mediating defective sensory-motor connectivity in SMA and to identify UBA1 dependent mediators of degeneration. We report that elevation of UBA1 levels via AAV9 gene therapy *in vivo* was sufficient to correct sensory-motor connectivity defects in SMA mice, acting via UBA1’s regulation of aminoacyl-tRNA synthetases, including GARS, in sensory neurons.

## Materials and methods

### Study approvals

All animal studies were approved by the internal ethics committee at the University of Edinburgh and were performed under the authority of relevant project and personal licenses from the UK Home Office.

### Animal models and UBA1 overexpression *in vivo*

Taiwanese SMA mice (*Smn*^−/−^; *SMN2^tg^*^/^*^0^*) (Hsieh-Li *et al.*, 2000; Riessland *et al.*, 2010), on a congenic FVB background, were originally obtained from Jackson Laboratories (strain no. 005058) and maintained according to established breeding protocols (Riessland *et al.*, 2010). Phenotypically-normal heterozygous (*Smn*^+/−^; *SMN2^tg^*^/^*^0^*) littermates were used as controls. Wild-type FVB mice were obtained from in-house breeding stocks at the University of Edinburgh. Mice were maintained under standard specific pathogen-free conditions and retrospectively genotyped using standard PCR protocols.

To overexpress UBA1 *in vivo*, mice were injected with AAV9-UBA1 intravenously on the day of birth (Powis *et al.*, 2016). AAV9-UBA1 (Vigene) was administered at a concentration of 7 × 10^13^ viral genomes. Mice underwent general chilled anaesthesia and were then injected with 10 µl of AAV9-UBA1 into the facial vein using a Hamilton syringe fitted with a Hamilton 33-gauge RN needle. Litters of mice were randomly assigned to treatment groups. For all subsequent analyses, the observer was blinded to the disease/treatment status of the mice.

### Tissue isolation and preparation

Mice were sacrificed by overdose of anaesthetic at postnatal Day 8, a late-symptomatic stage of disease progression. Spinal cord dissection was performed as previously described (Powis and Gillingwater, 2016). For quantitative fluorescent western blot analysis, isolated spinal cords were snap-frozen on dry ice and stored at −80°C. For immunohistochemistry, spinal cords were fixed in 4% paraformaldehyde (PFA) (Electron Microscopy Sciences) for 24 h and cryopreserved in 30% sucrose for 24 h at 4°C. Lumbar regions of spinal cord were embedded in O.C.T. (Cell Path), sectioned at 25 μm on a cryostat, immediately collected onto SuperFrost Plus™ microscope slides (Thermo Scientific) and stored at −20°C.

For immunohistochemistry of lumbar dorsal root ganglia (DRG), spinal columns were dissected from postnatal Day 8 mice, cut transversely at the T13 vertebrae, fixed for 6 h in 4% PFA, and cryopreserved in 30% sucrose for 24 h at 4°C. Lumbar and lower thoracic regions were embedded in O.C.T. and cryosectioned at 12 µm. Using the T13 DRGs, spinal columns were aligned so that the DRGs on each side of the column were symmetrical, sections from L1 and L2 DRGs were collected onto SuperFrost Plus™ microscope slides. For quantitative fluorescent western blot analysis lumbar dorsal root ganglia were dissected out of the spinal column using a previously described method (Sleigh *et al.*, 2016). For each mouse, three DRGs from each half of the lumbar spinal column were dissected so that western blots were performed on six DRGs per mouse.

### Cell culture and transfection

HEK293 cells (European Collection of Authenticated Cell Cultures) were grown in high glucose Dulbecco’s modified Eagle medium (Life Technologies) supplemented with 10% heat inactivated foetal bovine serum (FBS; Sigma), penicillin/streptavidin (Invitrogen) and l-glutamine (Invitrogen). HEK293 cells were transfected with RNAiMAX (Invitrogen) and 2.5 µM Silencer® Select Validated UBA1 siRNA (s601, targeted against exons 24 and 25; Life Technologies), or with Lipofectamine™ (Invitrogen) and 3.5 µg pCMV6-XL4-UBA1 plasmid (Origene) for proteomics samples. For proteomics, three separate transfections were combined per sample with three samples per condition. For co-transfection of multiple plasmids, 1.25 µg of each plasmid was used and the same amount of DNA and Lipofectamine™/RNAiMax was added to each well: pCMV6-XL4-UBA1 (Origene), pCMV6-Entry (Origene); pEGFP-GARS-N2 (gift from Dr Antonelli Antonellis), pEGFP-N1 (gift from Prof. Mike Cousin); pcDNA3.1-HA-Ubiquitin (gift from Prof. Yeh), Addgene plasmid #18712 (Kamitani *et al.*, 1997), pcDNA3.1+ (gift from Prof. Mike Cousin); pcDNA3.1-SMN-V5-His. Negative control 2 siRNA (Life Technologies) was used as a control for UBA1 siRNA. For all co-transfections, cells were harvested 48 h after transfection.

### Ubiquitylation assay

Following transfection, 10 µM of MG132 proteasome inhibitor was added to HEK293 cells for 90 min. Cells were lysed in RIPA buffer (Fisher Scientific) containing 1% protease inhibitor cocktail (Life Technologies), 1% phosphatase inhibitor (Life Technologies) and 10 mM *N*-ethylmaleimide (Sigma). The lysate was incubated with 0.5 µg anti-GFP primary antibody for 1 h 20 min at 4°C before incubation with protein A magnetic beads (Life Technologies) for 40 mins at 4°C. Immunoprecipitates were then washed four times in NP40 buffer. Immunoprecipitates and input controls were probed with the indicated antibodies.

### Protein extraction and western blotting

Protein was extracted in RIPA buffer with 1% protease inhibitor cocktail, homogenized using a motorized disposable pestle mixer. Protein concentration was determined using a BCA assay (ThermoScientific). Proteins were separated on a 4–12% gradient gel (Novex) and blotted using PVDF membrane stacks using the iBlot® system (Life Technologies). Western blotting was performed using primary antibodies against SMN (1:1000, BD Transduction Laboratories), UBA1 (1:1000, Thermo, PA5–17274), GARS (1:2500, Abcam, ab42905), YARS (1:1000, Abcam, ab154819), HA (1:1000, CST, 3724P), GFP (1:5000, Abcam, ab290) and the appropriate IRDye® secondary antibodies (LICOR). Tissue and cell-type appropriate loading controls were: histone H3 (1:5000, Abcam, ab1791), CoxIV (1:1000, Abcam, ab14744), α-Tubulin (1:5000, Abcam, ab7291), GAPDH (1: 2500, Abcam, ab9484) and Ponceau S total protein stain. Membranes were scanned on an Odyssey® imager (LICOR; Eaton *et al.*, 2013) and analysed using ImageStudio (LICOR).

### Immunohistochemistry

Spinal cord and spinal column sections were permeabilized in 0.3% Triton™ X-100 (Sigma) in PBS for 10 min at room temperature, blocked in 4% BSA, 0.1% Triton™ X-100 in PBS for 1 h and incubated in primary antibodies in a 1:4 dilution of blocking solution at 4°C for 24 h. Slides were washed in PBS and incubated with appropriate Alexa Fluor®-labelled secondary antibodies (1:400; Life Technologies) for 1 h at room temperature and subsequently in DAPI (Life Technologies) for 10 min. For immunohistochemistry of NF200, peripherin, parvalbumin and cleaved caspase 3, spinal column sections were thawed at room temperature for 1 h and permeabilized in 0.3% Triton™ X-100 in PBS (PBST) for 3 × 10 mins at room temperature, and blocked in 10% BSA in PBST for 1 h. Sections were then incubated with primary antibodies in blocking solution, at 4°C for 24 h. Slides were washed and sections incubated with the appropriate Alexa Fluor®-labelled secondary antibodies (1:400; Life Technologies) in PBS for 1 h at room temperature and incubated in DAPI for 10 min. All slides were mounted and coverslipped in a 10% Mowiol® solution (Polyscience). The following primary antibodies were used in this study: GARS, Abcam, ab42905, 1:500; UBA1a, CST, 4890S, 1:200; ChAT, Millipore, AB144P, 1:100; VGlut1, synaptic systems, 135 302, 1:1000; SMI32, Covance, SMI-32R, 1:1000; NF200, Sigma, N0142, 1:500; peripherin, Merck, AB1530, 1:500; parvalbumin, Swant, PV27, 1:1000; βIII-tubulin, Abcam, ab41489, 1:500; cleaved caspase-3, CST, 9661, 1:500.

### Imaging and analysis

Imaging was performed on a Nikon A1R FILM at the IMPACT facility, University of Edinburgh, or a Zeiss LSM710 confocal microscope. For distribution analysis, all imaging was performed at constant confocal settings. *Z*-stack images were taken and individual motor neurons selected for analysis based on an intact nucleus and an intact cell body, as ascertained by DAPI and ChAT, respectively; motor neurons were analysed in the plane where the nuclear outline was most clearly defined. To count the number of VGLUT1 synapses per ChAT-positive motor neuron soma, *Z*-stack images were taken with a slice interval of 0.2 µm and a pixel size of 0.08 µm throughout the whole section thickness to include the whole cell body (Mentis *et al.*, 2011). Whole DRGs were imaged in a single plane and selected for imaging and subsequent analysis based on structural integrity. Only sections that included a full cross-section through the centre of the DRG were imaged. One left and one right DRG were imaged per lumbar segment from L1 and L2 for each mouse. For distribution analysis of sensory neurons, seven larger area neurons and seven smaller area neurons were analysed per DRG. All example images of DRGs are from the L2 segment. Distribution analysis was performed in Fiji using the XOR and measurement functions (Ferreira and Rasband, 2012) to measure the intensity of the protein of interest in the nucleus and cytoplasm independent of each other (Supplementary Fig. 1) (Zhang *et al.*, 2015). Neuron area was measured by outlining the cell profile (NF200 or peripherin) in Fiji. Sensory neurons were only analysed if a clear nuclear outline was observed. The cell counter plugin in Fiji was used to count NF200- or peripherin-positive neurons (Sleigh *et al.*, 2017), and to count the number of VGLUT1 synapses per ChAT-positive soma.

### Label-free proteomics

HEK293 cell pellets were lysed in SDT lysis buffer containing 100 mM Tris-HCl (pH 7.6; Sigma), 4% (w/v) SDS (VWR). Protein concentration was determined using a BCA assay (ThermoScientific). Aliquots (100 μg) of each HEK293 lysate were processed through filter-aided sample preparation involving buffer exchange to 8 M urea and alkylation with 50 mM iodoacetamide. Double digestion with trypsin (Roche, sequencing grade) was then performed, initially for 4 h, then overnight, at 30°C. Samples were desalted by washes with 0.1% trifluoroacetic acid and then 70% acetonitrile. Each sample was separated by injection onto a nanoflow LC-MS/MS Ultimate 3000 RSLC (Thermo Scientific) system coupled to a linear ion trap Orbitrap hybrid mass spectrometer (LTQ Orbitrap Velos Pro, Thermo Scientific) via a nanoelectrospray ion source (Thermo Scientific). Peptides from each digest were separated with a linear gradient of 2–40% acetonitrile, 0.1% formic acid over 124 min with a constant flow of 300 nl/min. Full-scan mass spectrometry (MS) survey spectra were acquired in the LTQ Oribitrap with a resolution of 60 000; this was followed by IT-MS/MS scans for the 15 most intense peptide ions. Data were acquired using Xcalibur™ software. Alongside the nine samples, four quality control samples were processed, each of which consisted of a mixture of the nine samples.

### Proteomic data analysis

Raw proteomic data were imported into Progenesis for analysis of relative ion abundance and peptide characterization. The MS/MS output were converted into 2D representations for each sample; these were then aligned to a quality control samples with all alignment scores >90%. The data were subsequently filtered where all ions with a charge >5 were removed, as were features detected <23 min and >137 min. The runs for the different biological replicates of each condition were combined and statistical *P*-values were automatically generated for the peptides in Progenesis software through a one-way ANOVA on the ArcSinh transform of the normalized data. The peptides were then filtered and those with a *P*-value <0.05 or a power >0.8 were removed (Supplementary Fig. 1A). The remaining data were then exported for identification of individual proteins using the IPI-*Homo sapiens* database via Mascot Search Engine (V2.3.2) in which 712 proteins were identified; statistical analysis was carried out automatically in Mascot. Proteins identified in Mascot were then imported into Progenesis for filtering and further analysis. Peptide conflicts were removed and proteins were filtered to eliminate those with less than two unique peptides; proteins with a *P*-value < 0.05 or a fold-change <1.1 in either UBA1 overexpression compared to control or knockdown compared to control were removed (Supplementary Fig. 1A). This resulting dataset contained 222 proteins, which showed the largest significant variation in expression following modulation of UBA1 expression levels (Supplementary Fig. 1B and C). UBA1 itself was removed from the dataset for all subsequent analysis.

The Database for Annotation, Visualization and Integrated Discovery (DAVID) was used to identify enriched biological themes within the filtered proteomics dataset; functional annotation clustering was performed in which enriched terms are grouped together to identify enriched functional clusters. Modified Fischer’s exact *P*-values for each term are automatically calculated during this analysis; annotation terms that belong to the same proteins are then grouped together and assigned an enrichment score, which is the geometric mean of the *P*-values for all the terms in that cluster. The enrichment score is the −log_10_ of the *P*-value so an enrichment score of 1.3 is equivalent to a *P*-value of 0.05. This software was also used to characterize the functions associated with the protein clusters generated in BioLayout. BioLayout (http://www.biolayout.org) was used to identify UBA1-dependent clusters of proteins based on the expression of the proteins across UBA1 overexpression, control and UBA1 knockdown. The network graphs in this study were generated using a Pearson correlation (set to 0.98) and Markov clustering algorithm (Enright *et al.*, 2002). The expression profiles are displayed as mean normalized abundance (pareto scale) with standard error of the mean (SEM) for each condition. Ingenuity pathway analysis (IPA; www.qiagenbioinformatics.com/products/ingenuity-pathway-analysis/) was used as previously described (Wishart *et al.*, 2007). The significance of an association between the dataset and a canonical pathway was defined by a Fischer’s exact *P*-value (Savli *et al.*, 2008). A 1.1-fold change threshold filter was applied in IPA to each dataset analysed and only experimentally observed interactions were selected for each analysis.

### Statistical analyses

All statistical analyses were performed in Prism 6 (GraphPad). Statistical analyses were performed using unpaired two-tailed Student’s *t*-test or a one-way ANOVA with a Tukey’s multiple comparison test for parametric data, and a Mann-Whitney test or a Kruskal-Wallis test with Dunn’s multiple comparisons test for non-parametric data, as appropriate. Data are reported as mean ± SEM.

### Data availability

Raw proteomics data files from this study are freely available for download from http://dx.doi.org/10.7488/ds/2405.

## Results

### Restoration of UBA1 with AAV9-UBA1 rescues sensory-motor connectivity defects in spinal muscular atrophy mice

**Figure 1:**
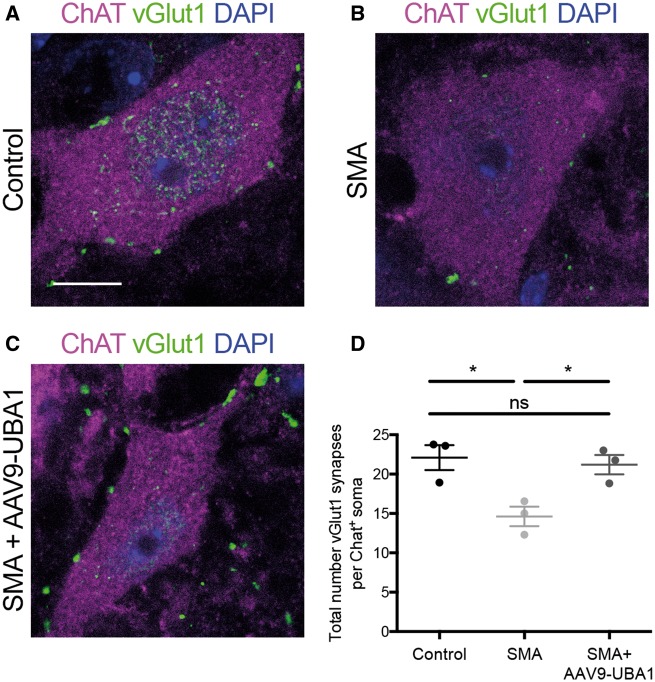
**Rescue of sensory-motor connectivity defects in the spinal cord of SMA mice following systemic restoration of UBA1.** (**A**–**C**) Lumbar motor neurons from control, SMA and SMA+AAV9-UBA1 mice labelled with ChAT (magenta), VGLUT1 (green) and DAPI. Scale bar = 10 µm. (**D**) Quantification of the number of VGLUT1 synapses per ChAT-positive motor neuron soma. *n* = 3 mice per condition, *n* > 20 motor neurons analysed per mouse. ns = not significant, *P < 0.05.

To establish whether UBA1-dependent pathways linked to SMA (Aghamaleky Sarvestany *et al.*, 2014; Hunter *et al.*, 2014; Wishart *et al.*, 2014; Fuller *et al.*, 2015; Powis *et al.*, 2016) contribute to the development of defective sensory-motor connectivity in SMA, we initially investigated the effects of systemic restoration of UBA1 on sensory-motor connectivity in SMA mice. Synaptic inputs formed onto lower motor neurons in the spinal cord were identified using immunohistochemical labelling of VGLUT1 (Ling *et al.*, 2010; Mentis *et al.*, 2011; Fletcher *et al.*, 2017; Simon *et al.*, 2017). Quantification of proprioceptive inputs onto lower motor neurons in the lumbar region of spinal cord revealed a significant reduction by 34% in symptomatic (postnatal Day 8) ‘Taiwanese’ SMA mice compared to littermate controls (Fig. 1A, B and D), confirming previous reports from different mouse models of SMA (Ling *et al.*, 2010; Mentis *et al.*, 2011). Systemic restoration of UBA1 levels, via intravenous delivery of AAV9-UBA1 on the day of birth (Powis *et al.*, 2016), completely restored the number of VGLUT1-positive synaptic inputs onto motor neurons in SMA mice (Fig. 1C and D). Thus, restoration of UBA1 was sufficient to ameliorate defective sensory-motor connectivity in SMA mice, suggesting that UBA1-dependent pathways play a significant role in the regulation of sensory neuron health *in vivo* and contribute directly to sensorimotor aspects of disease pathogenesis in SMA.

### Label-free proteomics identifies UBA1-dependent proteins

**Figure 2:**
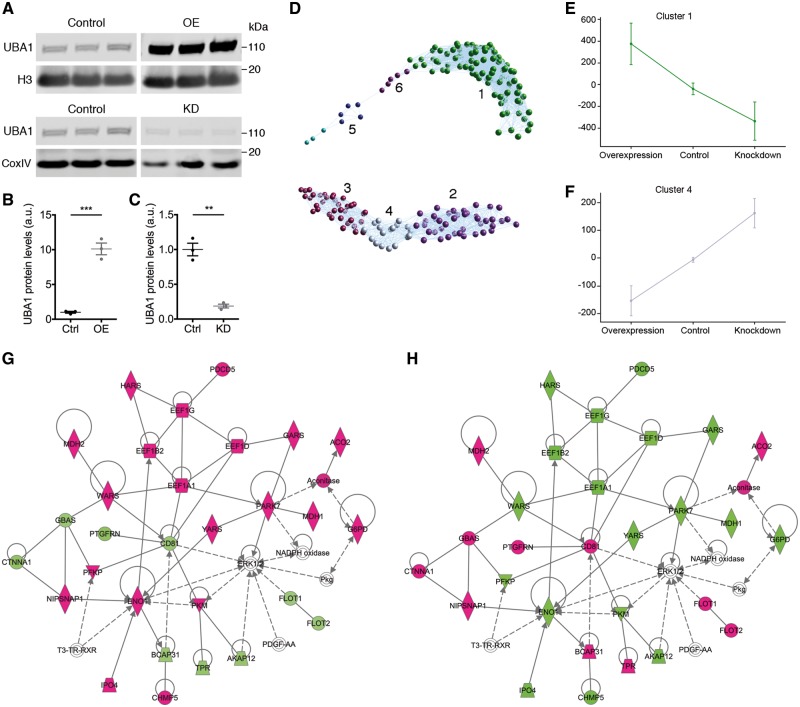
**Label-free proteomics analysis identifies tRNA synthetases as UBA1-dependent proteins.** (**A**) UBA1 expression in HEK293 cells used for proteomics screen showing UBA1 overexpression (OE) and knockdown (KD) compared to control. CoxIV and H3 = loading control. (**B** and **C**) Quantification of UBA1 overexpression (**B**) and knockdown (**C**). Ctrl = control; **P < 0.01, ***P < 0.001. (**D**) BioLayout clustering 3D representation of proteomic expression across UBA1 overexpression, control and UBA1 knockdown. Nodes represent proteins and edges represent similarity of expression between proteins and clusters of proteins with similar expression profiles are indicated by different colours. Cluster numbers are indicated. (**E** and **F**) Expression profile means ± SEM in log scale for the two UBA1-dependent clusters: cluster 1 (**E**) and cluster 4 (**F**). (**G** and **H**) IPA analysis showing the top network for UBA1 overexpression/control (**G**) and UBA1 knockdown/control (**H**), proteins highlighted in magenta are upregulated, proteins highlighted in green are downregulated. See also Supplementary Figs 2–5 and Supplementary Table 1.

To identify downstream targets of UBA1 likely to be mediating its effects on sensory-motor connectivity in SMA, label-free proteomics was performed on HEK293 cells where UBA1 had been either overexpressed (using a *UBA1* plasmid) or knocked down (using *UBA1* siRNA). HEK293 cells were chosen for the proteomics screen to enable dissection of UBA1 dependent pathways relevant for degeneration in SMA, independent of the reduction in SMN expression seen in SMA. Western blot confirmed a 10.11-fold increase in levels of UBA1 protein in cells overexpressing UBA1 (Fig. 2A and B) and a 76% reduction in UBA1 protein levels in cells transfected with *UBA1* siRNA (Fig. 2A and C). Following filtering of the raw proteomic data (Supplementary Fig. 2), 222 unique proteins were identified with a fold-change >10% after either overexpression or knockdown of UBA1. Of the proteins that revealed expression changes, the majority were upregulated following UBA1 overexpression and conversely downregulated following UBA1 knockdown (Supplementary Fig. 2).

Gene ontology term enrichment analysis was performed on the filtered proteomic data in DAVID, identifying aminoacyl-tRNA synthetases (also known as tRNA ligases), translation elongation factors, small molecule synthesis, and protein and RNA transport as significantly enriched gene ontology term clusters with the highest enrichment scores (Table 1). Gene ontology terms enriched with lower enrichment score included ubiquitin-like conjugation, protein complex assembly, and ATP and nucleotide binding (Table 1); functions related to the ubiquitin conjugation process. A similar analysis performed in IPA confirmed RAN signalling (a process involved in nuclear transport) and tRNA charging (a function of aminoacyl-tRNA synthetases) as significantly enriched canonical pathways. Nine of the 39 proteins involved in the tRNA charging canonical pathway were present in this dataset (Supplementary Table 1).

**Table 1:** Gene ontology term enrichment of proteins changed following modulation of UBA1 expression

GO term cluster	Enrichment score
Translation elongation	4.56
tRNA ligase activity	4.31
Nucleic acid synthesis	4.10
Cytoskeleton	3.30
Protein transport and localisation	3.03
RNA transport and localisation	2.95
Protein complex assembly	2.85
ATP and nucleotide binding	2.55
Glycolysis	2.45
Cytoskeletal binding	2.36
Ubl conjugation	2.25
Membrane bound vesicle	2.04
GTPase binding	2.02

See also Supplementary Table 1.

To identify specific UBA1 target proteins, BioLayout analyses were performed whereby clusters of proteins were generated based on the similarity of protein expression profiles. This analysis revealed two main groups of proteins modified downstream of UBA1, each of which was further subdivided, generating six subclusters of proteins (Fig. 2D). Two of the clusters (clusters 1 and 4) showed UBA1-dependent expression profiles (Fig. 2E and F), while the other four clusters showed a prominent expression change in only one experimental condition (Supplementary Fig. 3). The first UBA1-dependent cluster of proteins, cluster 1, contained proteins that were upregulated following UBA1 overexpression and downregulated following UBA1 knockdown (Fig. 2E). Subsequent analysis in DAVID revealed that the proteins in this cluster function as aminoacyl-tRNA synthetases or in glycolysis, translation elongation or assembly of protein complexes. Cluster 4 showed the opposite UBA1-dependent expression profile (downregulation following UBA1 overexpression and upregulations following UBA1 knockdown; Fig. 2F) in which proteins function in transport and localization of protein, RNA and nucleic acids. Interestingly, the functions of the UBA1-dependent clusters overlap with the enriched gene ontology terms and canonical pathways (Table 1 and Supplementary Table 1); thus, indicating that these are key protein families and functions changed on modulation of UBA1 protein levels.

To investigate UBA1 dependency at the level of individual proteins and interaction networks, a network analysis was performed using IPA software. Interestingly, the top protein network generated for each condition (overexpression or knockdown of UBA1) was composed of the same proteins, in which 25 of 30 proteins present in the network behaved in a UBA1-dependent manner (Fig. 2G and H; see Supplementary Figs 4 and 5 for expanded networks), suggesting that modulation of UBA1 expression causes widespread reciprocal changes in expression in discrete networks of proteins.

Importantly, our analyses repeatedly highlighted aminoacyl-tRNA synthetases (including GARS, YARS, HARS and WARS) as key UBA1 downstream targets (Fig. 2G and H). This finding was of particular interest and relevance due to known links between this family of enzymes and neurodegenerative conditions, with mutations in several of these genes causing hereditary neuropathies (Kapur *et al.*, 2017). More specifically, the GlyRS protein (GARS) encoded by *GARS* was of particular interest because of its known role in Charcot-Marie-Tooth disease type 2D (CMT2D), a neuromuscular disorder with phenotypic similarity to SMA (Antonellis *et al.*, 2003; Sivakumar *et al.*, 2005; James *et al.*, 2006; Motley *et al.*, 2010).

### UBA1 regulates GARS expression independent of its role in ubiquitylation

**Figure 3:**
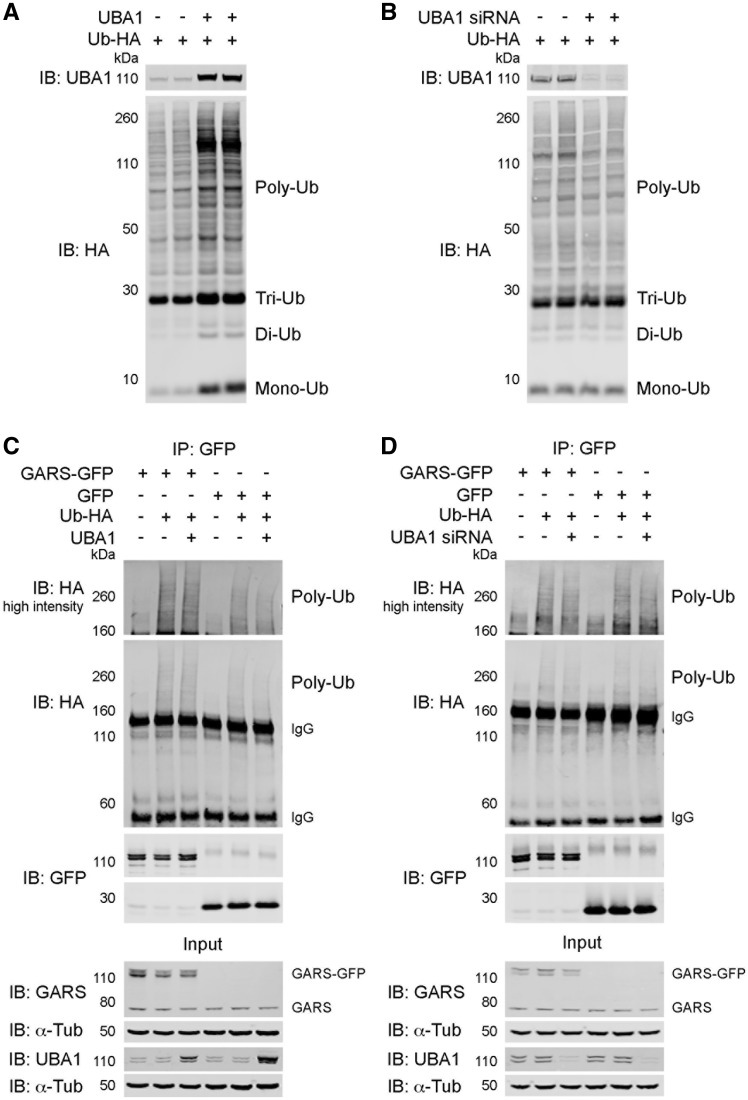
**UBA1 influences GARS expression through a non-canonical function.** (**A** and **B**) HEK293 cells were transfected with ubiquitin-HA (Ub-HA) and UBA1 (**A**) or UBA1 siRNA (**B**); western blot of UBA1 and ubiquitin (immunoblotted for HA tag), showing polyubiquitylated substrate proteins (Poly-Ub), and free triubiquitin (Tri-Ub), diubiquitin (Di-Ub) and monoubiquitin (Mono-Ub). (**C** and **D**) HEK293 cells were transfected with GARS-GFP or GFP along with Ub-HA and UBA1 (**C**) or UBA1 siRNA (**D**). Input control samples were immunoblotted for UBA1 and GARS; immunoprecipitation (IP) with GFP; IP samples were immunoblotted for GFP and HA. IgG bands and polyubiquitylation (Poly-Ub) smears are indicated. High intensity immunoblots show polyubiquitylation smears imaged at increased laser power. See also Supplementary Fig. 6.

As the canonical role of UBA1 concerns priming of the ubiquitylation cascade (Groen and Gillingwater, 2015), we next sought to investigate whether the downstream protein targets of UBA1 identified in our proteomics screen were showing different levels of expression due to direct perturbations in their ubiquitylation status. To investigate the effect of UBA1 modulation on overall ubiquitylation levels, HEK293 cells were transfected with ubiquitin (Ub-HA) and a *UBA1* plasmid, *UBA1* siRNA or a suitable control vector. As expected, following overexpression of UBA1 overall polyubiquitylation of substrate proteins was increased (Fig. 3A). Following UBA1 knockdown, there was a concomitant, albeit less prominent, change in overall ubiquitylation status with a modest reduction in polyubiquitylation of substrate proteins (Fig. 3B). Overall, this confirms that modulation of UBA1 levels leads to changes in ubiquitylation of substrate proteins, demonstrating that UBA1 can influence the expression of downstream targets through differential ubiquitylation.

To investigate whether the UBA1-dependent tRNA-synthetase GARS was being differentially ubiquitylated following modulation of UBA1 expression, a protein-specific ubiquitylation assay was performed. As the ubiquitylation pattern of SMN is well characterized (Chang *et al.*, 2004; Kwon *et al.*, 2013; Abera *et al.*, 2016), the ubiquitylation assay, in which the proteins of interest were co-transfected, was initially optimized using SMN, confirming the ubiquitylation pattern of SMN (Supplementary Fig. 6). Surprisingly, following overexpression of UBA1, in the presence of both GARS and ubiquitin, there was no change in the overall ubiquitylation of GARS (Fig. 3C). Similarly, following UBA1 knockdown, there was also no change in the ubiquitylation of GARS compared to control UBA1 expression (Fig. 3D). Thus, modulation of UBA1 expression did not affect the ubiquitylation of GARS, suggesting that UBA1 influences the expression of GARS through a non-canonical pathway independent of its role in the ubiquitylation cascade.

### Perturbations in UBA1 lead to disruption of GARS in spinal muscular atrophy mice

**Figure 4:**
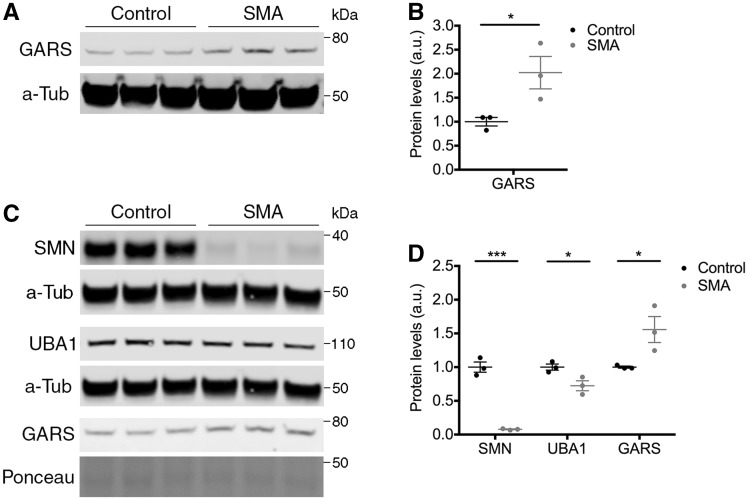
**GARS is dysregulated in neuronal tissue from SMA mice.** (**A** and **B**) Western blot (**A**) and quantification (**B**) of GARS protein levels in spinal cord from late-symptomatic SMA mice and control littermates. α-Tubulin (α-Tub) = loading control. (**C** and **D**) Western blot (**C**) and quantification (**D**) of SMN, UBA1 and GARS protein levels in DRG from late-symptomatic SMA mice and control littermates. α-Tubulin (α-Tub), total protein (Ponceau): loading controls. *n = *3 mice per condition. See also Supplementary Figs 7 and 8.

To explore the potential role and relevance of the UBA1/GARS pathway in SMA pathogenesis further, we next wanted to establish whether GARS protein was disrupted in SMA *in vivo.* Furthermore, to investigate the wider effects of UBA1 on tRNA synthetases, we quantified the expression of the tRNA-synthetase YARS in SMA mice; mutations in *YARS* cause dominant intermediate CMT type C (DI-CMTC) (Jordanova *et al.*, 2006). Importantly, it has previously been demonstrated that both UBA1 and SMN expression are reduced in spinal cord from Taiwanese SMA mice (Powis *et al.*, 2016; Groen *et al.*, 2018*a*). GARS showed an increase of 100% in spinal cord from SMA mice at postnatal Day 8 compared to control littermates (Fig. 4A and B) whilst YARS showed a reduction of 37% in SMA spinal cord (Supplementary Fig. 7A and B); thereby validating expression changes in two different tRNA-synthetases in SMA.

Interestingly, however, there was no significant difference in GARS expression specifically within lower motor neuron bodies in the spinal cord (Supplementary Fig. 8A and B), suggesting that modulations in UBA1/GARS pathways are unlikely to underlie motor neuron pathology in SMA. In stark contrast, expression changes of GARS were present in the dorsal root ganglia (DRG), which contain sensory neuron bodies (Fig. 4C and D). As protein levels of SMN and UBA1 have not previously been reported for DRGs in control and SMA mice, their expression was quantified by western blot analysis. As expected, a significant reduction of SMN was observed in DRGs (Fig. 4C) with an overall reduction of 92% in SMA mice compared to controls (Fig. 4D). This is consistent with the reduction of SMN protein reported in SMA patients (Lefebvre *et al.*, 1997) and the reduced levels of *Smn* transcript reported in DRGs from ‘delta7’ SMA mice (Mentis *et al.*, 2011). Similarly, UBA1 was reduced by 28% in late-symptomatic SMA DRGs compared to controls (Fig. 4C and D), consistent with the magnitude of UBA1 reduction reported in spinal cord from late-symptomatic Taiwanese SMA mice (Powis *et al.*, 2016). Consistent with the change in expression seen in spinal cord, GARS expression was upregulated by 56% in SMA DRGs compared to controls (Fig. 4C and D), while YARS expression showed no change in SMA DRGs (Supplementary Fig. 7A and B). Thus, suppression of UBA1 downstream of SMN leads to a significant increase in GARS protein levels in SMA mice *in vivo*, with DRGs being particularly affected.

### Identification of a sensory neuron fate phenotype in spinal muscular atrophy mice

The increased levels of GARS we observed in SMA were consistent with changes in GARS protein levels reported in CMT2D, where they are notably increased during neonatal development and disease progression (Achilli *et al.*, 2009; Motley *et al.*, 2010, 2011). It has recently been shown that disruption to sensory neuron fate is a significant feature of disease pathogenesis in CMT2D mice, leading to an alteration in the functional subpopulations of sensory neurons in the DRG (Sleigh *et al.*, 2017). We therefore wanted to establish whether similar GARS-dependent phenotypes were contributing to the sensory-motor connectivity defects that occur in SMA.

Large area sensory neurons within DRGs are predominantly either mechanoreceptors (sense touch) or proprioceptors (sense position and movement), both of which stain positive for NF200: ∼20% of NF200-positive neurons are proprioceptors and the remaining 80% are mechanoreceptors (Sleigh *et al.*, 2017). Smaller area sensory neurons stain positive for peripherin and are mostly nociceptive. In CMT2D mice there is a severity-dependent disruption to the fate of sensory neurons, with a decrease in the percentage of NF200-positive neurons and an increase in the percentage of peripherin-positive neurons, leading to concordant behavioural deficits (Sleigh *et al.*, 2017).

**Figure 5:**
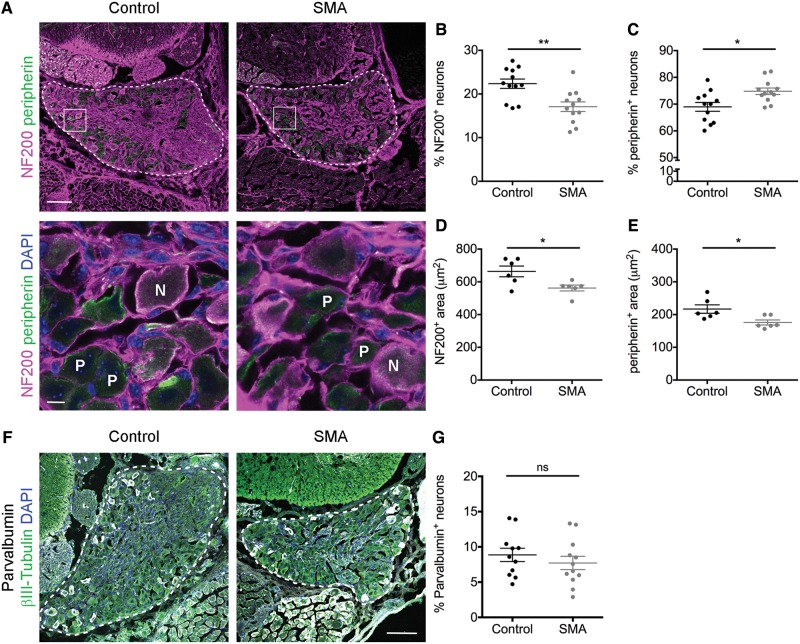
**Sensory neuron fate is disrupted in SMA dorsal root ganglia.** (**A**) Spinal column sections from lumbar segments 1 and 2 of late-symptomatic SMA and control mice labelled with NF200 (magenta) and peripherin (green). DRG are outlined in the *top* panels, boxes indicate the area in *bottom* panel. Nuclei of cells in *bottom* panels are labelled with DAPI. N = NF200-positive neurons; P = peripherin-positive neurons. Scale bars = 100 µm (*top*), 10 µm (*bottom*). (**B** and **C**) Quantification of the percentage of NF200-positive (NF200^+^) (**B**) and peripherin-positive (peripherin^+^) (**C**) sensory neurons. *n* = 3 mice per condition, *n = *4 DRGs per mouse. (**D** and **E**) Quantification of the area of NF200^+^ (**D**) and peripherin^+^ (**E**) sensory neurons. *n* = 3 mice per condition, *n = *2 DRGs per mouse (seven NF200^+^ and seven peripherin^+^ neurons per DRG were analysed). (**F**) Spinal column sections from lumbar segments 1 and 2 of late-symptomatic SMA and control mice labelled with parvalbumin (white), beta-III tubulin (green) and DAPI (blue), dorsal root ganglia (DRG) are outlined. Scale bar = 100 µm. (**G**) Quantification of parvalbumin-positive sensory neurons as a percentage of beta-III tubulin-positive sensory neurons. *n* = 3 mice per condition, *n = *4 DRGs per mouse. *P < 0.05, **P < 0.01. See also Supplementary Fig. 9.

Qualitatively, DRGs from SMA mice appeared smaller with fewer NF200-positive neurons compared to control mice (Fig. 5A and B). Quantification revealed a significant 24% reduction in the percentage of NF200-positive neurons relative to control (Fig. 5B), with a concomitant increase in peripherin-positive neurons by 85% relative to control (Fig. 5C). CMT2D mice have a reduction in the area of sensory neuron cell bodies (Sleigh *et al.*, 2017). Likewise, NF200-positive neurons were significantly smaller in SMA mice than in controls, with an average reduction of 101 µm^2^ per neuron (Fig. 5D). Similarly, peripherin-positive neurons were also significantly smaller in SMA mice (Fig. 5E), thereby confirming that SMA DRGs were indeed smaller than those in control animals, as has previously been reported for other organs in SMA (Powis *et al.*, 2016; Thomson *et al.*, 2017; Maxwell *et al.*, 2018). Despite the overall reduced size and disruption to sensory neuron fate in SMA DRGs, these phenotypes were not due to overt cell death in the SMA DRGs (Supplementary Fig. 9), a finding consistent with previous reports in CMT2D (Sleigh *et al.*, 2017).

Because of the identification of a reduction in the proportions of NF200-positive sensory neurons in SMA, we next wanted to investigate whether mechanoreceptors and proprioceptors were differentially affected. Quantification of the overall percentage of sensory neurons that stain positive for parvalbumin, a proprioceptor specific marker, revealed no significant difference in the proportion of parvalbumin-positive sensory neurons in SMA DRGs compared to control (Fig. 5F and G). This is consistent with a previous report in a different mouse model of SMA (Mentis *et al.*, 2011), and demonstrated that, as in CMT2D (Sleigh *et al.*, 2017), both subtypes of NF200-positive sensory neurons were equally affected leading to the disruption to sensory neuron fate seen in SMA.

Taken together, these findings reveal a novel sensory neuron fate phenotype associated with increased levels of GARS in SMA mice, highlighting significant pathological overlap between SMA and CMT2D at the level of sensory neurons.

### Dysregulation of UBA1/GARS-dependent pathways in sensory neurons from spinal muscular atrophy mice

**Figure 6:**
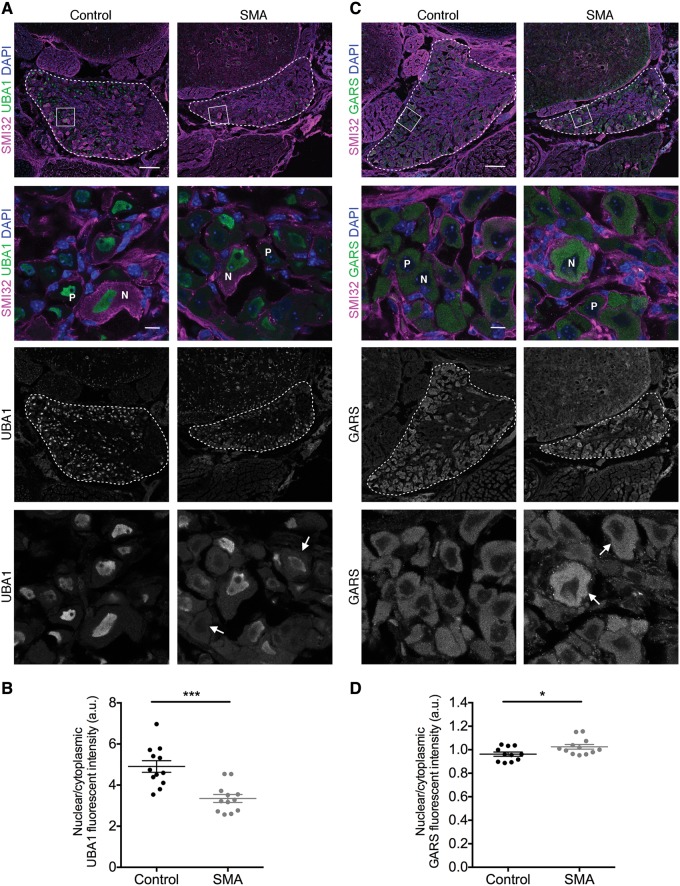
**Dysregulation of UBA1 and GARS in dorsal root ganglia sensory neurons from SMA mice.** (**A** and **C**) Spinal column sections from lumbar segments 1 and 2 of late-symptomatic SMA and control mice labelled with UBA1a (**A**) or GARS (**C**) (green), SMI32 (magenta) and DAPI. DRG are outlined in *overview* panels, boxes indicate the area in *lower* panels. Arrows in *bottom* panel indicate sensory neurons with altered UBA1 (**A**) or GARS (**C**) expression. N = NF200-positive neurons; P = peripherin-positive neurons. Scale bars = 100 µm (*overview*), 10 µm (*lower* panels). (**B** and **D**) Late-symptomatic SMA DRG sensory neurons show a reduction in the nuclear to cytoplasmic ratio of UBA1 (**B**) and an increase in the nuclear to cytoplasmic ratio of GARS (**D**) compared to control mice. *n* = 3 mice per condition, *n = *4 DRGs per mouse (14 sensory neurons were analysed per DRG); *P < 0.05, ***P ≤ 0.001.

To investigate links between UBA1/GARS-dependent pathways and disrupted sensory neuron fate in SMA further, the ratio of UBA1 and GARS expression was investigated at the level of individual sensory neurons within the DRG. There was less prominent nuclear labelling and more pronounced cytoplasmic labelling of UBA1 in DRGs from SMA mice, indicating a shift in the subcellular distribution of UBA1 (Fig. 6A). Quantification revealed a significant reduction of the nuclear-cytoplasmic ratio (NCR) by 24% in SMA mice compared to controls (control = 5.34, SMA = 4.07; Fig. 6B). Sensory neurons also showed a significant increase in the NCR of GARS in SMA compared to controls (Fig. 6C and D). Notably, however, larger diameter neurons revealed a selective increase in the intensity of GARS labelling (Fig. 6C). Interestingly, here changes in GARS expression occurred in the opposite direction to changes in UBA1 expression, consistent with changes at the whole tissue level as detected by western blot analysis of whole DRGs and spinal cord (Fig. 4). Thus, sensory neuron cell fate phenotypes observed in SMA mice correlate directly with parallel expression changes in key proteins of the UBA1/GARS pathway.

### Restoration of UBA1 rescues sensory neuron fate phenotypes in spinal muscular atrophy mice

**Figure 7:**
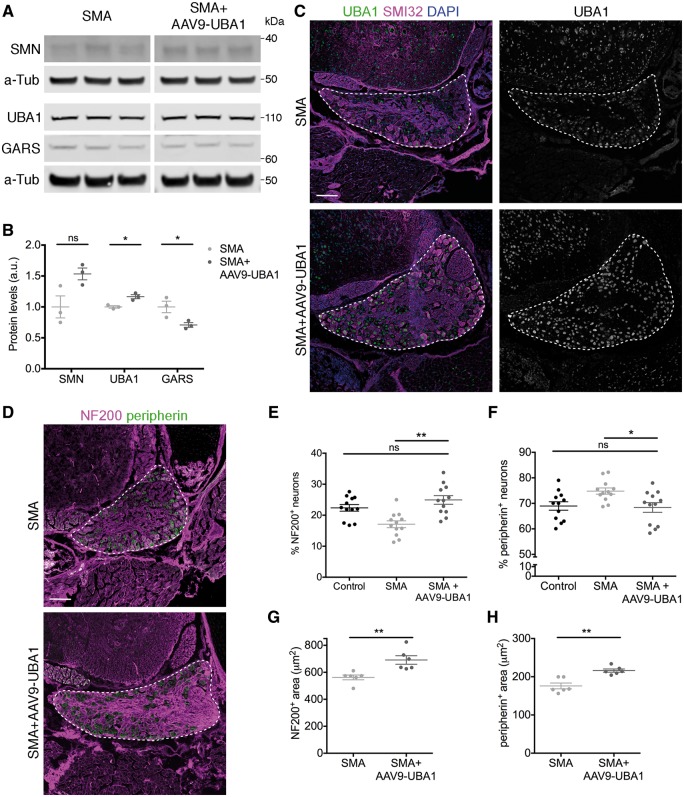
**Restoration of UBA1 in SMA mice reverses GARS dysregulation and rescues sensory neuron cell fate phenotypes.** (**A** and **B**) Representative fluorescent western blot (**A**) and quantification (**B**) of SMN, UBA1 and GARS in dorsal root ganglia from late-symptomatic SMA mice and SMA mice injected with AAV9-UBA1 (SMA+AAV9-UBA1). α-Tubulin (α-Tub): loading control. *n = *3 mice per condition. (**C**) Spinal column sections from lumbar segments 1 and 2 of late-symptomatic SMA and SMA+AAV9-UBA1 mice labelled with UBA1a (green), SMI32 (magenta) and DAPI. (**D**) Spinal column sections from lumbar segments 1 and 2 of late-symptomatic SMA and SMA+AAV9-UBA1 mice labelled with NF200 (magenta) and peripherin (green). (**C** and **D**) Dorsal root ganglia (DRG) are outlined. (**E** and **F**) Quantification of the percentage of NF200-positive (NF200^+^) (**E**) and peripherin-positive (peripherin^+^) (**F**) sensory neurons. Data from control mice is shown as reference. *n* = 3 mice per condition, *n = *4 DRGs per mouse. (**G** and **H**) Quantification of the area of NF200^+^ (**G**) and peripherin^+^ (**H**) sensory neurons. *n = *3 mice per condition, *n = *2 DRGs per mouse (seven NF200^+^ and seven peripherin^+^ neurons per DRG were analysed); ns = not significant. *P < 0.05, **P < 0.01. See also Supplementary Figs 10 and 11.

Finally, we wanted to establish whether the rescue of sensory-motor connectivity observed in the spinal cord of SMA mice treated with AAV9-UBA1 was due to correction of sensory neuron fate phenotypes in the DRG and if this disruption to sensory neuron fate in SMA mice was directly dependent on changes in the UBA1/GARS pathway. Quantitative western blot performed on lumbar DRGs from FVB mice and FVB mice injected with AAV9-UBA1 confirmed transduction of DRG sensory neurons, with an increase in UBA1 expression by 33% in mice injected with AAV9-UBA1, but no change in SMN expression (Supplementary Fig. 10A and B). Immunofluorescence confirmed transduction of sensory neurons at the cellular level (Supplementary Fig. 10C). Similarly, SMA mice and SMA mice injected with AAV9-UBA1 revealed a significant increase in UBA1 levels, by 17% compared to non-injected animals at postnatal Day 8, but no change in SMN expression (Fig. 7A and B). Upregulation of UBA1 was also evident at the cellular level within individual sensory neurons of the DRG (Fig. 7C). Importantly, upregulation of UBA1 using AAV9-UBA1 reduced GARS levels by 29% (Fig. 7A and B), back toward the levels observed in DRGs from control mice.

To determine whether AAV9-UBA1-mediated correction of GARS levels rescued sensory neuron fate phenotypes, we assessed sensory neuron subtype proportions in SMA mice and SMA mice injected with AAV9-UBA1. Overexpression of UBA1 in SMA mice fully rescued the disruption to sensory neuron fate (Fig. 7D) whereby the percentage of NF200-positive neurons was increased by 46% in SMA mice injected with AAV9-UBA1 relative to SMA mice (Fig. 7E). There was also a significant reduction in the percentage of peripherin-positive neurons in SMA mice injected with AAV9-UBA1 by 9% relative to SMA mice (Fig. 7F). Compared to data obtained from control mice, there was no significant difference in the percentage of NF200- or peripherin-positive neurons in SMA mice treated with AAV9-UBA1 (Fig. 7E and F). Interestingly, following treatment with AAV9-UBA1 in SMA mice there was a significant increase in the percentage of parvalbumin-positive neurons, from 8% of the total sensory neuron population in SMA mice, to 13% in SMA mice injected with AAV9-UBA1 (Supplementary Fig. 11A and B). This indicates that AAV9-UBA1 predominantly affects proprioceptive sensory neurons.

Overexpression of UBA1 in SMA mice also rescued the area of sensory neurons with an increase in the area of NF200-positive neurons by an average of 129 μm^2^ per neuron (Fig. 7G) and an increase in peripherin-positive neuron area by an average of 40 μm^2^ in treated SMA mice (Fig. 7H). Thus, restoration of UBA1 levels using viral gene delivery in SMA mice was sufficient to correct GARS protein levels in sensory neurons and fully rescue the disruption to sensory neuron fate.

## Discussion

Taken together, data from the current study demonstrate that UBA1 is a major regulator of sensory-motor connectivity phenotypes in SMA. Aminoacyl-tRNA synthetases were identified as key UBA1 downstream targets, revealing a novel UBA1/GARS dependent pathway that mediates sensory neuron cell fate through a non-canonical pathway distinct from differential ubiquitylation. Restoration of UBA1 in SMA mice using a gene therapy approach revealed that correction of UBA1 levels, and downstream UBA1/GARS pathways, was sufficient to rescue sensory neuron cell fate phenotypes, and sensory-motor connectivity defects in the spinal cord, in SMA mice (Supplementary Fig. 12). Because of the known role of mutant GARS in mediating sensory neuron pathology in CMT2D, our findings provide experimental evidence of a significant molecular and phenotypic overlap between SMA and CMT.

The finding that the newly-identified UBA1/GARS pathway was regulated independently of the ubiquitylation status of GARS adds further support to a growing body of evidence suggesting that UBA1 can influence its downstream targets and pathways through non-canonical functions. For example, functions of UBA1 outside of the canonical ubiquitylation cascade have previously been reported in Atg8-dependent autophagy (Chang *et al.*, 2013). Our findings therefore extend our understanding of the broad range of pathways influenced by UBA1, and confirm the existence of multiple, non-canonical functions for UBA1 *in vivo.* Consistent with this model, recent studies have suggested that UBA6 ubiquitylates a larger proportion of the proteome than previously thought and indicated that ubiquitylation of GARS may be performed by UBA6 (Liu *et al.*, 2017*b*).

Moreover, the tissue-specific nature of UBA1-dependent perturbations in GARS and YARS identified here echoes previous work in which downstream molecular targets of UBA1 were found to vary depending on the tissue and organ being examined (Wishart *et al.*, 2014). For example, therapies treating the accumulation of β-catenin downstream of UBA1 in SMA, rescued neuromuscular but not systemic/non-neuromuscular pathologies in Taiwanese SMA mice (Wishart *et al.*, 2014). Furthermore, it has previously shown that AAV9-UBA1 increases SMN expression in hearts from both SMA and control mice compared to untreated SMA and control mice, respectively (Powis *et al.*, 2016). Here, we report that AAV9-UBA1 does not increase SMN expression in either SMA (Fig. 7A and B) or control DRGs (Supplementary Fig. 10A and B), indicating that modest SMN restoration may contribute to the rescue of some SMA phenotypes, but not the restoration of sensory neuron phenotypes following AAV9-UBA1 treatment. This suggests that therapies restoring UBA1, which lead to widespread rescue of SMA phenotypes (Powis *et al.*, 2016), are likely to be modulating numerous, tissue-specific downstream pathways of UBA1, thereby revealing a complex spatiotemporal influence of ubiquitin pathways on disease pathogenesis in SMA and related conditions.

While sensory neuron pathology has previously been identified in models of SMA (Jablonka *et al.*, 2006; Ling *et al.*, 2010; Mentis *et al.*, 2011; Fletcher *et al.*, 2017), the disruption to sensory neuron fate and reduction in the size of sensory neurons within the DRG reported here provides important insights into the pathogenesis of sensory-motor connectivity defects in SMA. A recent study suggested that motor neuron hyperexcitability in SMA occurs specifically due to defects in proprioceptive sensory neurons, with the onset of motor neuron dysfunction correlating with sensory-motor connectivity defects in SMA. Importantly, blocking neurotransmission specifically in proprioceptive neurons in wild-type mice caused severe motor defects, shortened lifespan and rendered motor neurons dysfunctional (Fletcher *et al.*, 2017). Together this suggests that restoration of proprioceptive synapses in SMA leads to increased functionality of motor neurons. Our finding that defects in sensory neuron fate specifically target NF200-positive neurons in SMA, and that restoration of UBA1 was sufficient to rescue sensory neuron fate in the DRG alongside sensory-motor connectivity in the spinal cord, suggests that UBA1/GARS-dependent regulation of this sensory neuron subtype plays a critical role in the pathogenesis of both motor and sensory phenotypes in SMA. It also highlights the possibility that UBA1/GARS-mediated restoration of sensory-motor connectivity leads to improvements in motor neuron function which, along with rescue of other motor neuron and muscle specific defects through alternative UBA1-mediated pathways, cause the phenotypic improvements in neuromuscular pathology previously reported in AAV9-UBA1 treated SMA mice (Powis *et al.*, 2016).

Importantly, sensory pathology in SMA patients is becoming increasingly well characterized (Supplementary Table 2). Several reports of patients with SMA type 1 indicate abnormal sensory conduction (Duman *et al.*, 2013) or absence of sensory responses (Reid *et al.*, 2016), as well as axonal degeneration in sensory nerves (Rudnik-Schoneborn *et al.*, 2003). In earlier studies of presumed SMA cases, axonal degeneration of sensory neurons was also identified, alongside ballooned neurons and chromatolysis within DRGs (Marshall and Duchen, 1975; Carpenter *et al.*, 1978; Murayama *et al.*, 1991). Furthermore, a genetically-confirmed case of XL-SMA showed nodules within the DRG, indicating a loss of sensory neurons (Supplementary Table 2) (Dlamini *et al.*, 2013).

Our finding of a key role for GARS in regulating sensory neuron phenotypes and sensory-motor connectivity in SMA, provides experimental support for the hypothesis that there is significant molecular and phenotypic overlap between SMA and CMT2D. Furthermore, the finding of altered YARS expression in SMA spinal cord indicates a wider overlap of spinal muscular atrophies and peripheral neuropathies. Interestingly, several cases of co-segregation of SMA and CMT have been reported (Supplementary Table 2) (Jedrzejowska *et al.*, 2008; Fernandez *et al.*, 2016), indicating that the pathologies of these two diseases can occur simultaneously. In addition to this, several genes have now been identified where mutations can cause a range of phenotypes incorporating both CMT and SMA-like diseases. *GARS* is one such gene, where mutations can cause CMT2D, distal-SMA type V or more classical infantile SMA (Supplementary Table 2) (Antonellis *et al.*, 2003; James *et al.*, 2006; Eskuri *et al.*, 2012). Similarly, mutations in *MORC2* can cause CMT type 2Z or SMA (Schottmann *et al.*, 2016; Sevilla *et al.*, 2016) and mutations in *IGHMBP2* have been shown to cause both CMT type 2S and SMA with respiratory distress type 1 (SMARD1; Supplementary Table 2) (Grohmann *et al.*, 2001; Cottenie *et al.*, 2014; Pedurupillay *et al.*, 2016; Liu *et al.*, 2017*a*). Moreover, mutations in *LRSAM1*, an E3 ubiquitin ligase, cause CMT type 2G (Peeters *et al.*, 2016) thereby implicating ubiquitin pathways in the pathogenesis of CMT. Similarly, mutations in *PIEZO2*, which encodes a mechanosensitive ion channel responsible for mechanosensation of light touch and proprioception, can cause a neuromuscular disease characterized by muscle atrophy, mild sensory involvement, delayed motor milestones and scoliosis (Chesler *et al.*, 2016; Delle Vedove *et al.*, 2016); suggesting that defects within sensory neurons themselves can lead to muscle defects and delayed motor development. Together, not only does this highlight considerable overlap between the phenotypes of CMT and SMA, but it also demonstrates considerable overlap of genetic pathways and molecular causes of these diseases, hinting at the potential of shared therapeutic opportunities for these neuromuscular conditions.

## Supplementary Material

Supplementary Figures and Tables

## Abbreviations

AAV9: adeno-associated virus serotype 9
CMT: Charcot-Marie-Tooth disease
DRG: dorsal root ganglion
SMA: spinal muscular atrophy
SMN: survival motor neuron protein
