# Supplementary material for: UBA1/GARS-dependent pathways drive sensory-motor connectivity defects in spinal muscular atrophy
Source: Brain. 2018 Sep 25;141(10):2878–94. doi: 10.1093/brain/awy237 (PMC6158753; doi:10.1093/brain/awy237)
Supplement: Supplementary Figures and Tables [file awy237_supplementary_figures-tables.pdf]

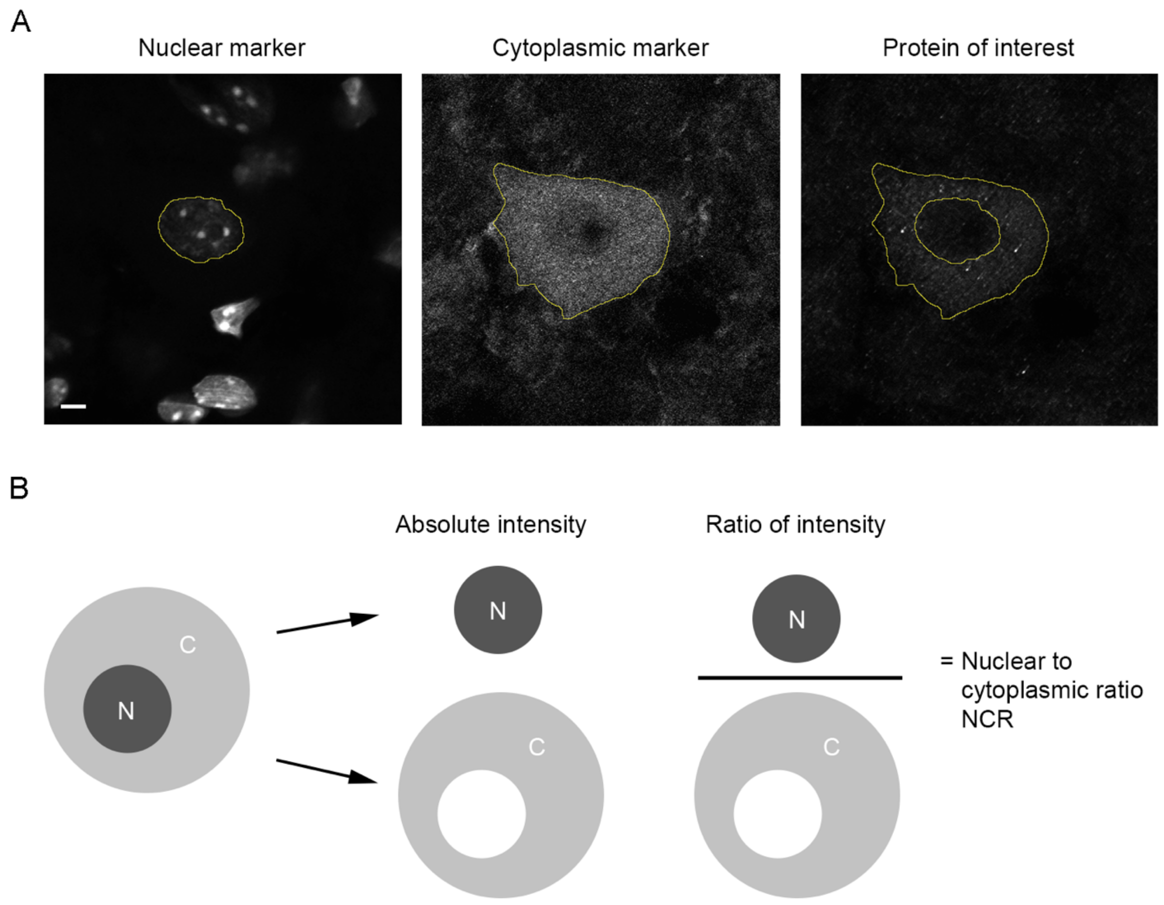

Figure S1

Intensity and distribution analysis

(**A**) Nuclear (DAPI) and cytoplasmic (ChAT) markers were used to outline the nucleus and cell body respectively (yellow lines). These outlines were then imposed onto the channel of the protein of interest. Scale bar: 5µm. (**B**) The XOR function was used so that the intensity of the protein of interest labelling could be measured separately in the nucleus and the cytoplasm. The intensity can then be presented as the absolute intensity for each cellular compartment or the ratio of nuclear to cytoplasmic intensity (NCR).

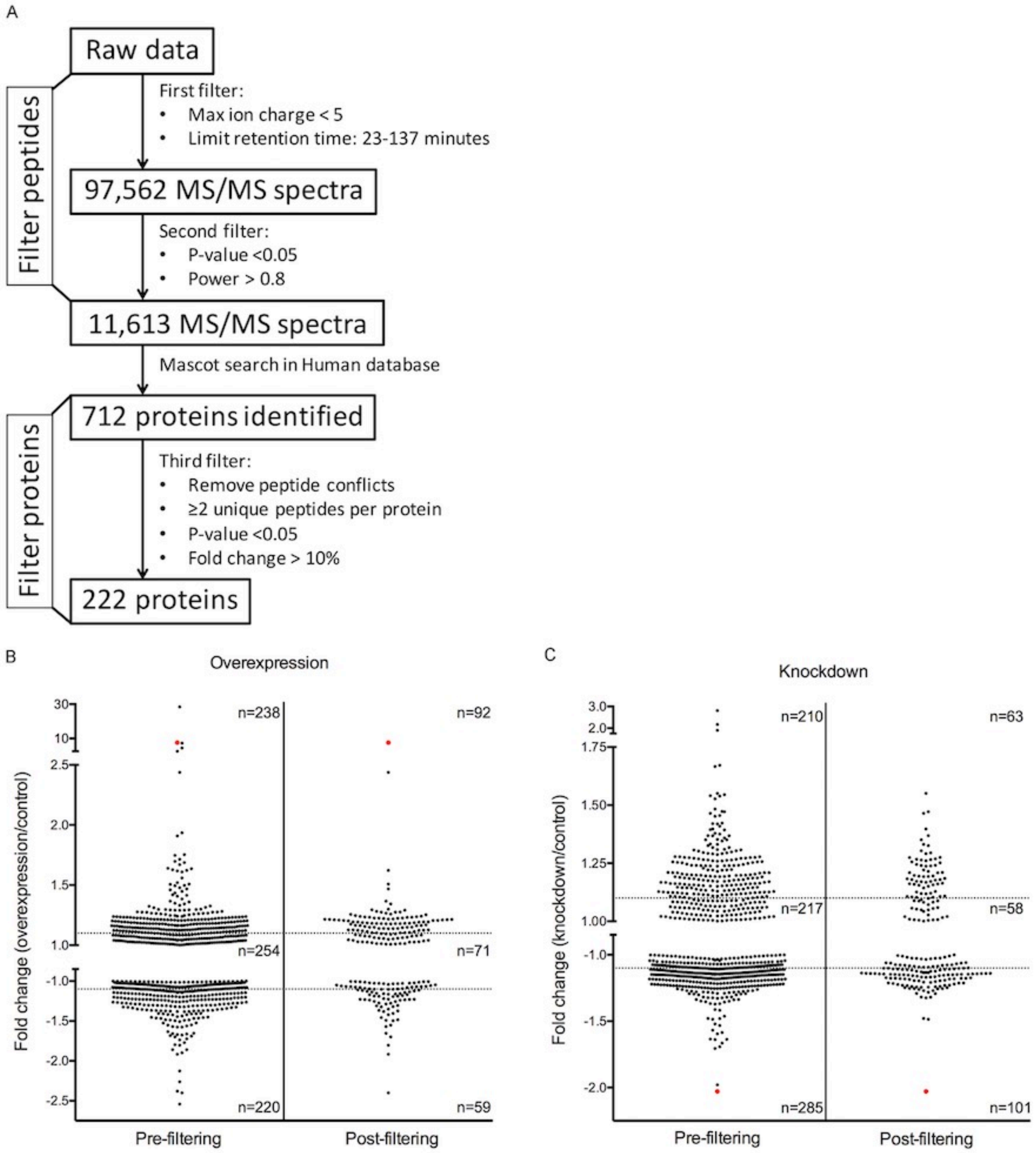

Figure S2

Filtering of proteomic data

(**A**) Filtering steps for raw proteomic data and proteins once identified in Mascot. (**B**) Fold-change of proteins identified following overexpression and (**C**) knockdown of UBA1 both pre- and post-filtering. Number of proteins with fold-change greater or less than +/-10% are indicated. Following UBA1 overexpression 68.02% of proteins detected showed changes in expression; following UBA1 knockdown, 73.87% of proteins detected showed changes in expression. UBA1 is shown in red and was removed for subsequent analysis so as not to skew the data.

Canonical pathway	p-value	Overlap	Number of proteins
RAN Signalling	2.76E-12	47.1%	8/17
Glycolysis I	1.14E-10	32.0%	8/25
Gluconeogenesis I	1.14E-10	32.0%	8/25
tRNA Charging	2.00E-10	23.1%	9/39
Purine Nucleotides De Novo Biosynthesis II	5.32E-10	54.5%	6/11

Table S1

IPA analysis reveals tRNA Charging as an enriched canonical pathway

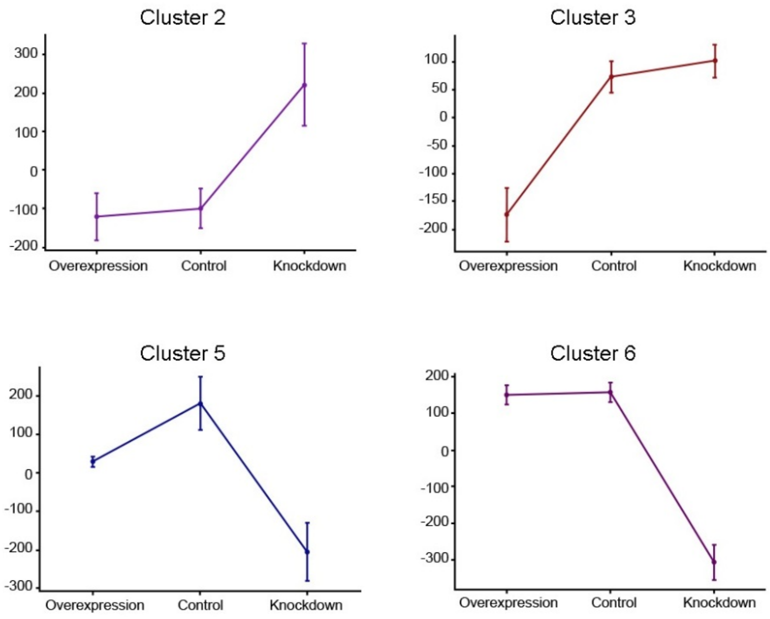

Figure S3

Protein clusters changed following UBA1 overexpression or UBA1 knockdown

Expression profile means with SEM in log scale for clusters that do not show UBA1 dependency, clusters 2, 3, 5 and 6.

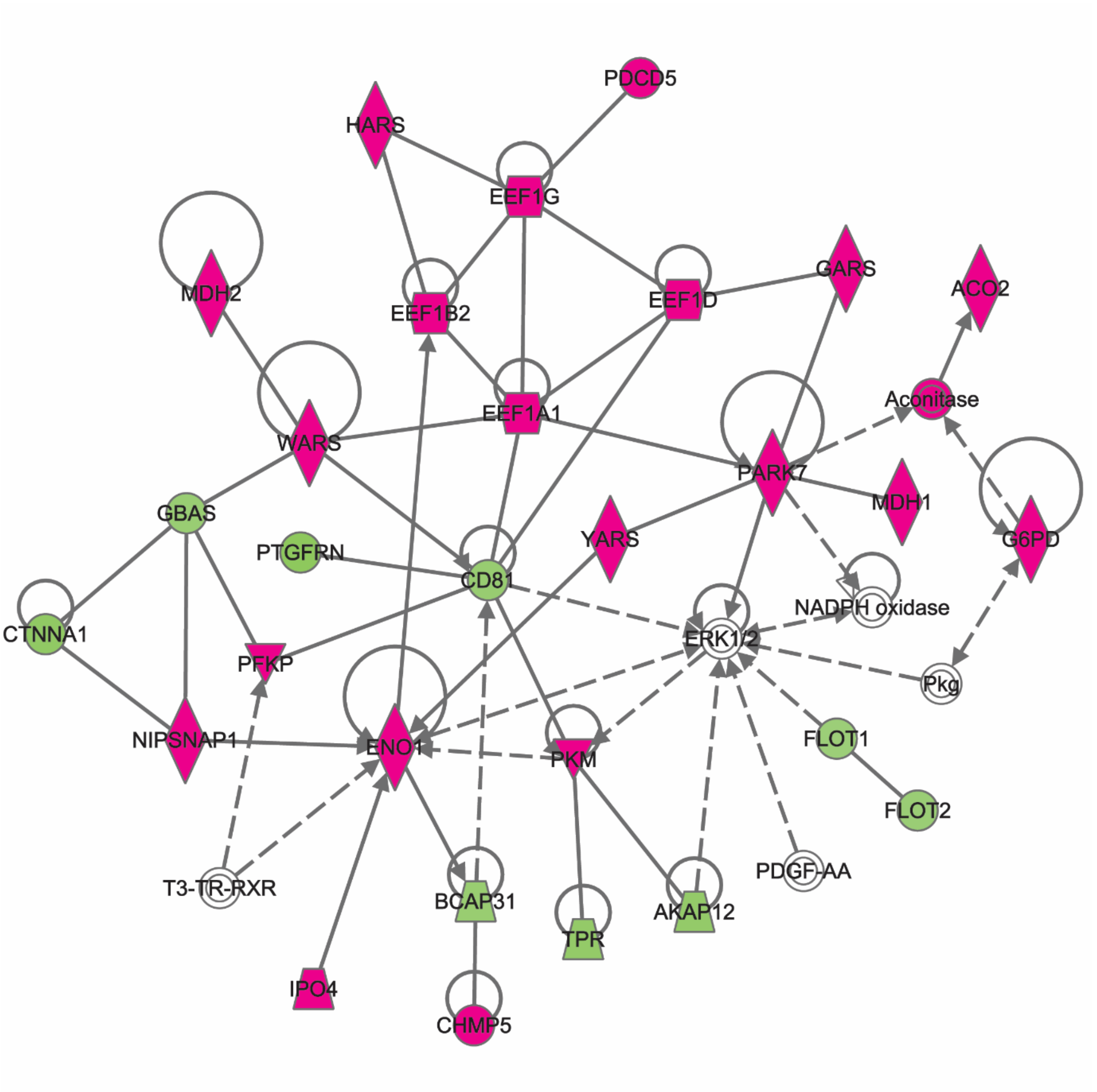

Figure S4

IPA top network for UBA1 overexpression/control. Proteins within magenta shapes are upregulated, proteins in green shapes are downregulated, proteins in white shapes are not present in the dataset. The shape of the protein in the network depicts the protein function: diamonds represent enzymes, triangles represent kinases, hexagons indicate translation regulators, trapeziums represent transporters, circles indicate other protein classes and circles with an inner circle depict a protein complex. Solid lines show direct binding of proteins, dotted straight lines show indirect interaction by protein binding. Solid arrow heads indicate when a protein acts on another protein and clear arrow heads show when a protein translocates to another protein or complex. Both networks contain the same proteins, most proteins show opposite expression changes in the two networks.

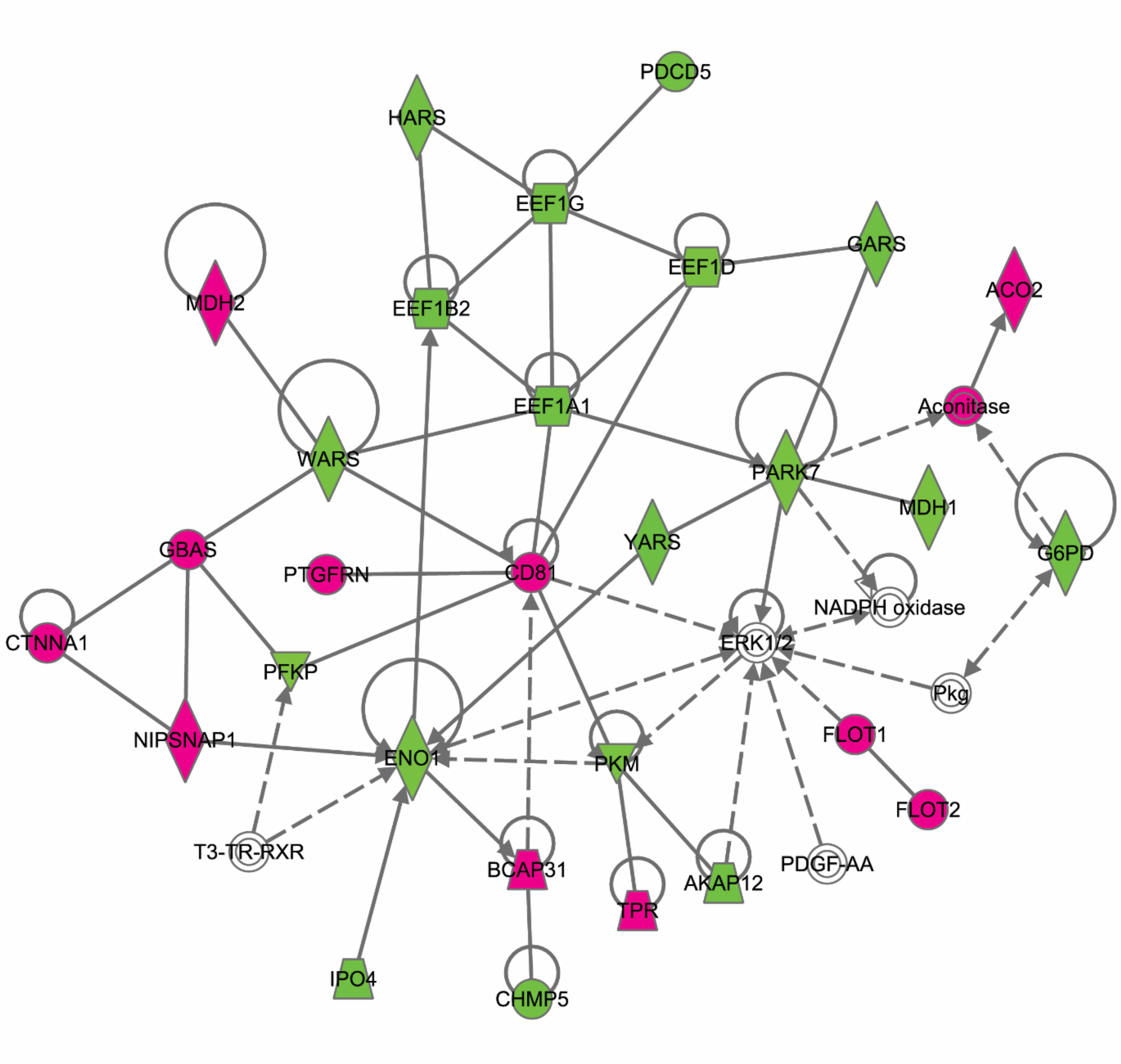

Figure S5

IPA top network for UBA1 knockdown/control. Proteins within magenta shapes are upregulated, proteins in green shapes are downregulated, proteins in white shapes are not present in the dataset. The shape of the protein in the network depicts the protein function: diamonds represent enzymes, triangles represent kinases, hexagons indicate translation regulators, trapeziums represent transporters, circles indicate other protein classes and circles with an inner circle depict a protein complex. Solid lines show direct binding of proteins, dotted straight lines show indirect interaction by protein binding. Solid arrow heads indicate when a protein acts on another protein and clear arrow heads show when a protein translocates to another protein or complex. Both networks contain the same proteins, most proteins show opposite expression changes in the two networks.

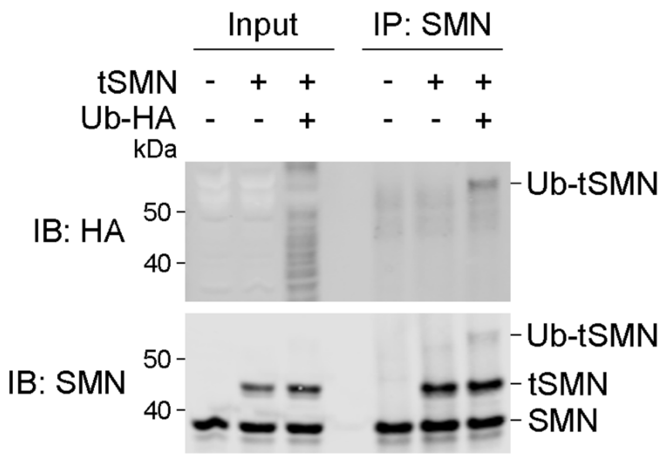

Figure S6

Ubiquitylation of SMN

Ubiquitylation of SMN can be reliably detected by performing a ubiquitylation assay. HEK293 cells were transfected with a tagged SMN construct (tSMN) and Ub-HA or control vector, SMN was immunoprecipitated (IP) and immunoblotted (IB) for both SMN and HA to detect ubiquitylation of the immunoprecipitated protein, in this case SMN; Ub-tSMN indicates ubiquitylated tSMN. kDa: molecular weight.

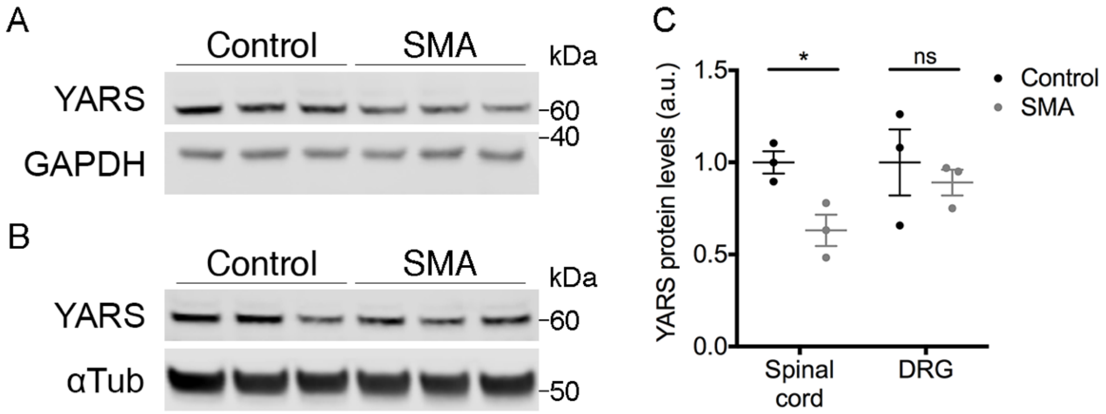

Figure S7

YARS expression is altered in SMA spinal cord but not in dorsal root ganglia

(**A** and **B**) Representative fluorescent Western blot of YARS protein levels in (**A**) spinal cord and (**B**) dorsal root ganglia. GAPDH and a-Tubulin (a-Tub) as loading controls. kDa: molecular weight. (**C**) Quantification of YARS protein levels in late-symptomatic SMA mice, n=3 mice per condition. ns – not significant, * $P \leq 0.05$.

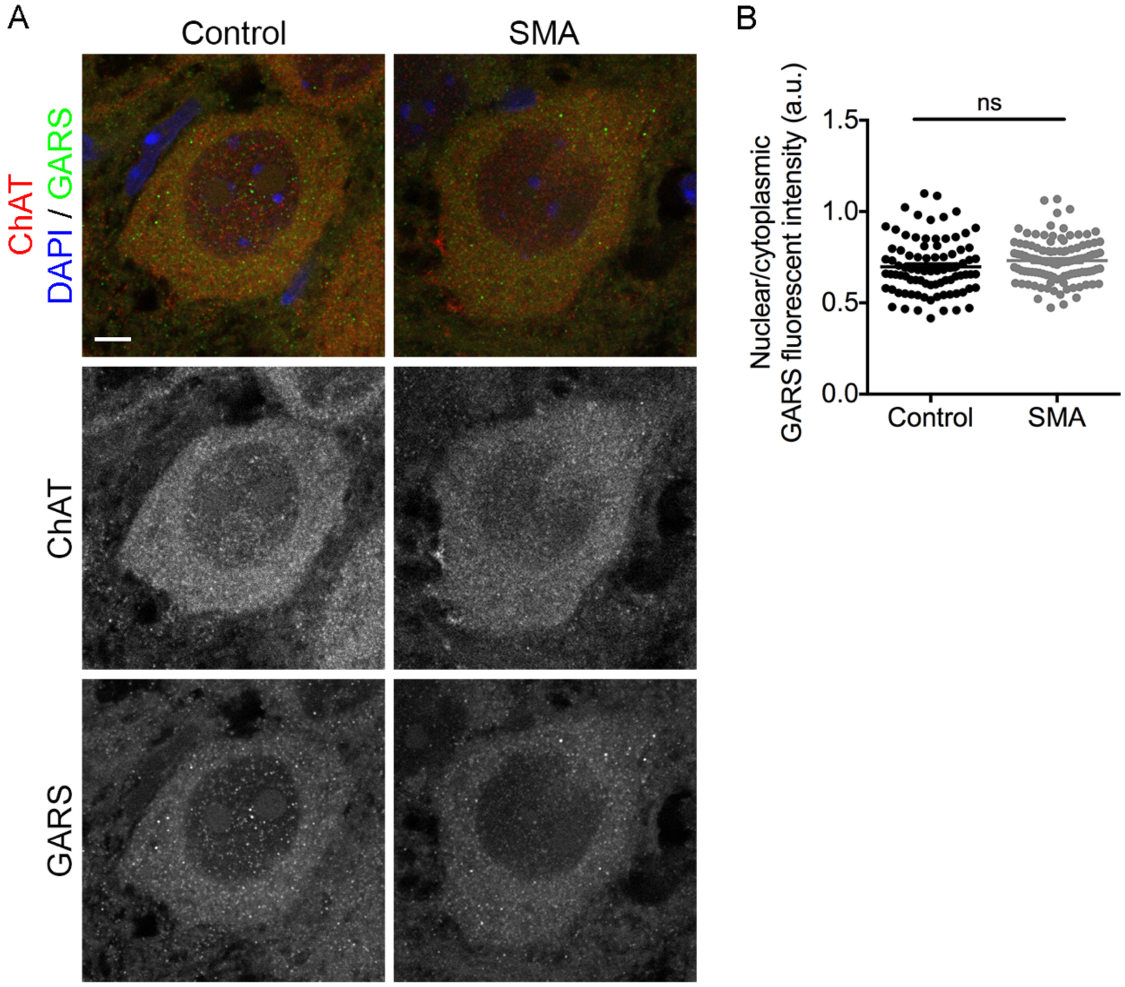

Figure S8

GARS expression is not disrupted in SMA spinal motor neurons

(**A**) Lower motor neurons in lumbar spinal cord sections from control and late symptomatic SMA mice labelled with GARS, ChAT and DAPI. Scale bar = 5µm. (**B**) Quantification of fluorescent intensity of the nuclear/cytoplasmic ratio of GARS. Control N=3 mice, n=85 motor neurons; SMA N=3 mice, n=106 motor neurons, ns - not significant.

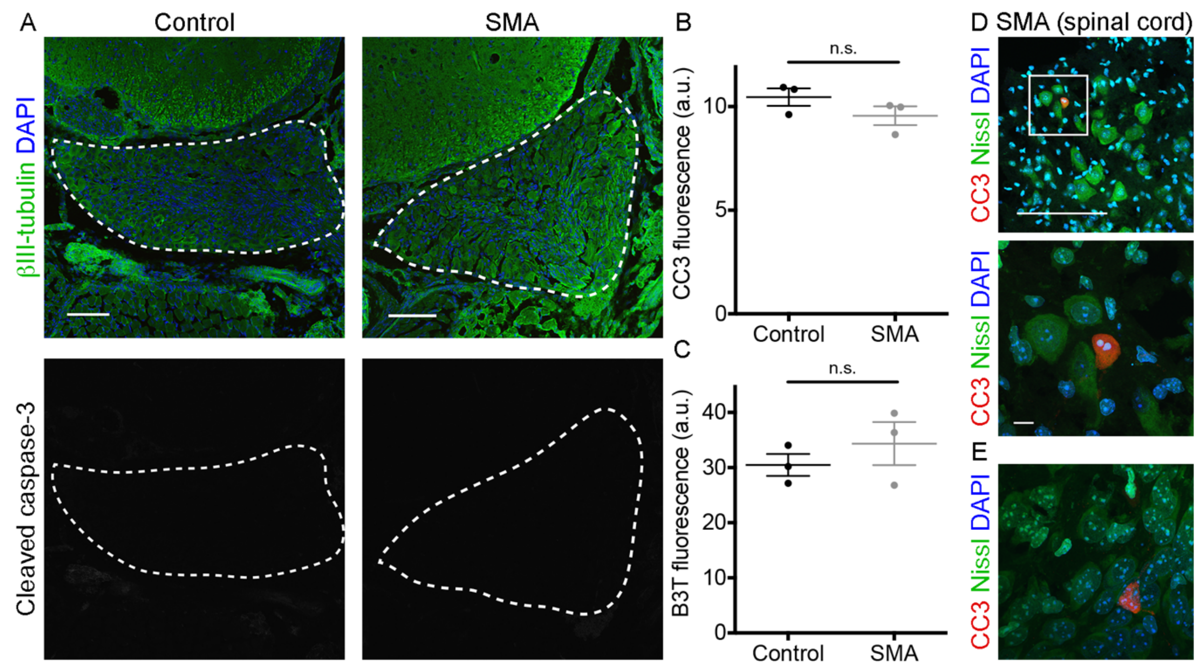

Figure S9

Cleaved caspase-3 immunostaining of SMA and control DRGs identified no evidence for sensory neuron death in SMA mice

(**A**) Representative images of control (left panels) and SMA (right panels) DRGs labelled with βIII-tubulin (B3T) and DAPI (green and blue respectively, top panels) and cleaved caspase-3 (CC3: greyscale, bottom panels). Scale bars = 100 µm. (**B**) Quantification of CC3 immunoreactivity identified no significant difference in SMA DRGs compared to control. (**C**) Quantification of B3T immunoreactivity identified no significant difference in SMA DRGs compared to control, indicating comparable DRGs were analysed between the two conditions. (**B** and **C**) N=3 mice per condition, n=2 DRGs per mouse, a.u.- arbitrary units, n.s.- not significant (P>0.05). (**D** and **E**) To confirm the validity of the CC3 antibody, we performed CC3 immunostaining on spinal cord sections from P8 SMA mice using the same protocols and experimental conditions as in **A**; spinal cord was counterstained with Nissl and DAPI to label neuronal cell bodies and nuclei, respectively. This identified several CC3-positive cells (**D** and **E**): inset (**D**, top panel) indicates high magnification of this neuron shown in the bottom panel of **D**. Aberrant nuclear morphology that be observed in CC3-postive neurons indicates active apoptotic / degenerative processes in CC3-positive neurons. Scale bar: 100 µm (**D** top panel), 10 µm (**D** bottom panel and **E**).

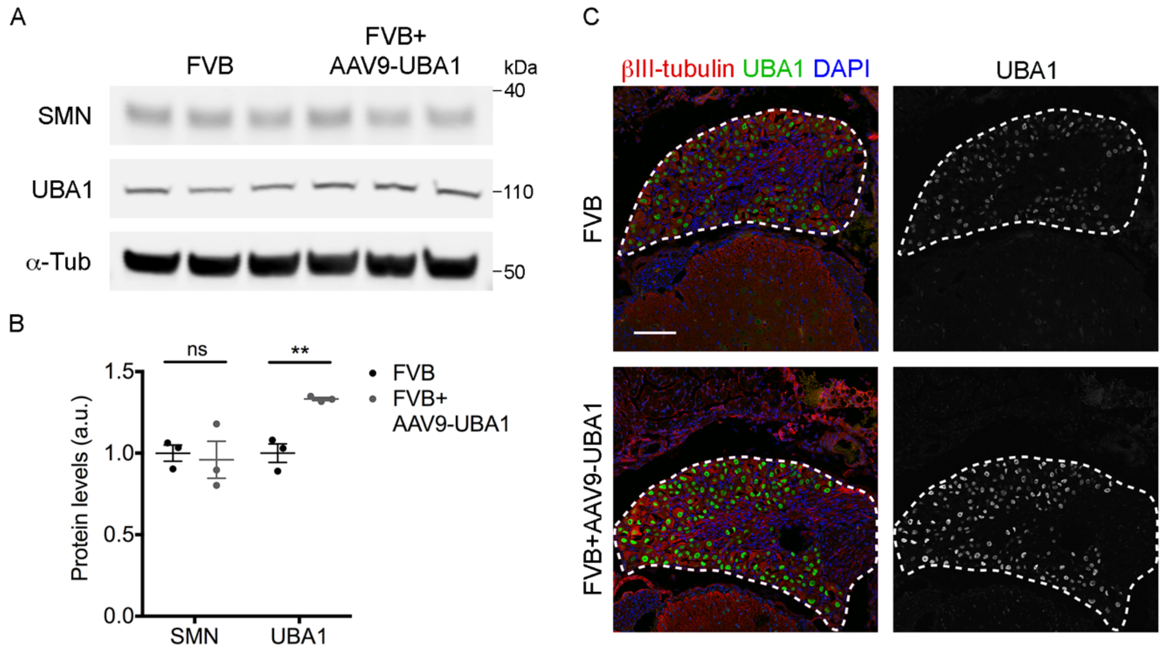

Figure S10

Systemic injection of AAV9-UBA1 transduces dorsal root ganglia sensory neurons.

(**A** and **B**) Representative fluorescent Western blot (**A**) and quantification (**B**) of SMN and UBA1 in dorsal root ganglia from AAV9-UBA1 injected wild-type FVB mice and littermate controls, a-Tubulin (a-Tub) as loading control. kDa: molecular weight; n=3 mice per condition; ns – not significant, ** $P \leq 0.01$; n=3 mice per condition. (**C**) Spinal column sections from lumbar segments 1 and 2 of late-symptomatic SMA and SMA+AAV9-UBA1 mice labelled with UBA1a (green), SMI32 (red) and DAPI, dorsal root ganglia are outlined. Scale bar = 100 µm.

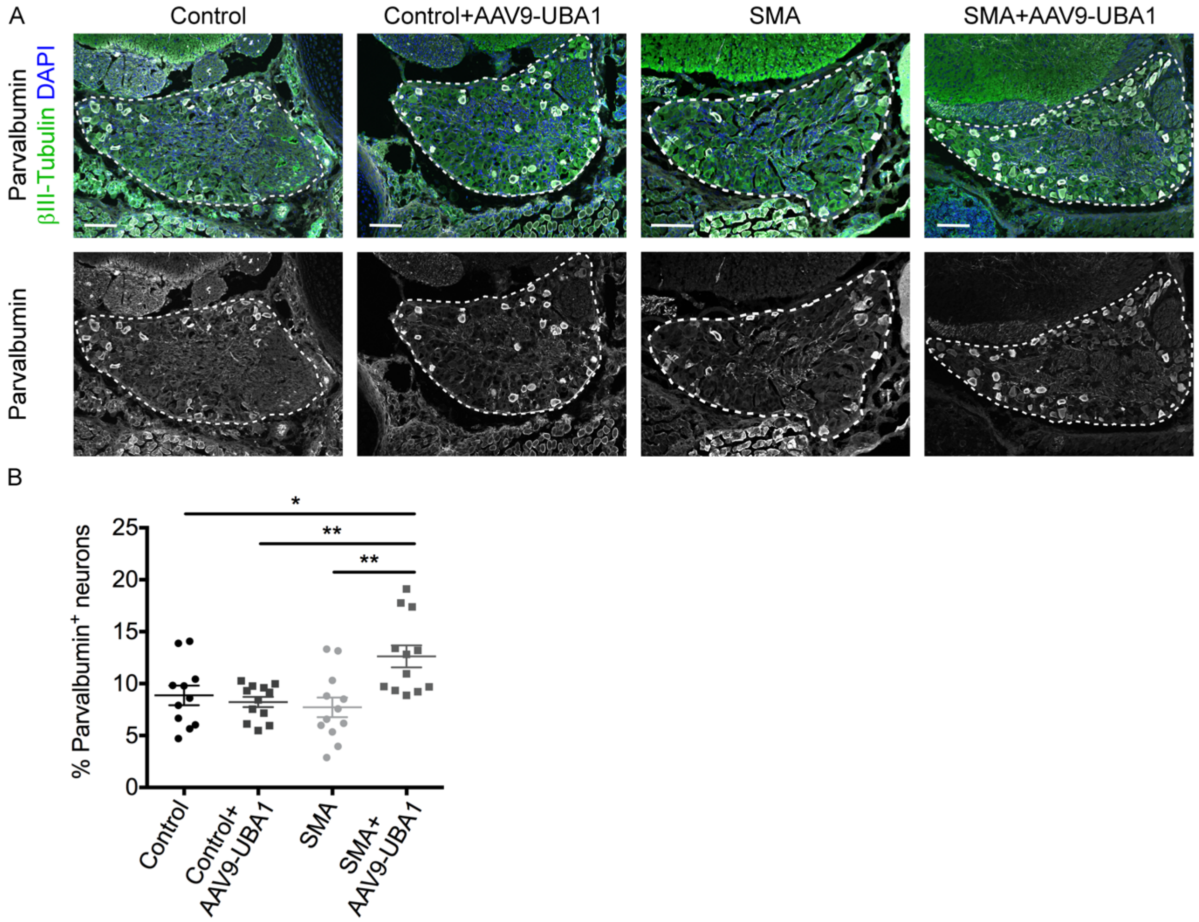

Figure S11

Overexpression of UBA1 *in vivo* increases the number of parvalbumin$^+$ sensory neurons

(**A**) Spinal column sections from lumbar segments 1 and 2 of late-symptomatic SMA and control mice labelled with parvalbumin (white), beta-III tubulin (green) and DAPI (blue), dorsal root ganglia (DRG) are outlined. Scale bars = 100µm. (**B**) Quantification of parvalbumin positive sensory neruons as a perentage of beta-III tubulin positive sensory neurons. N=3 mice per condition, n=4 DRGs per mouse. Control and SMA data replicated from Figure 5. * $p<0.05$, ** $p<0.01$.

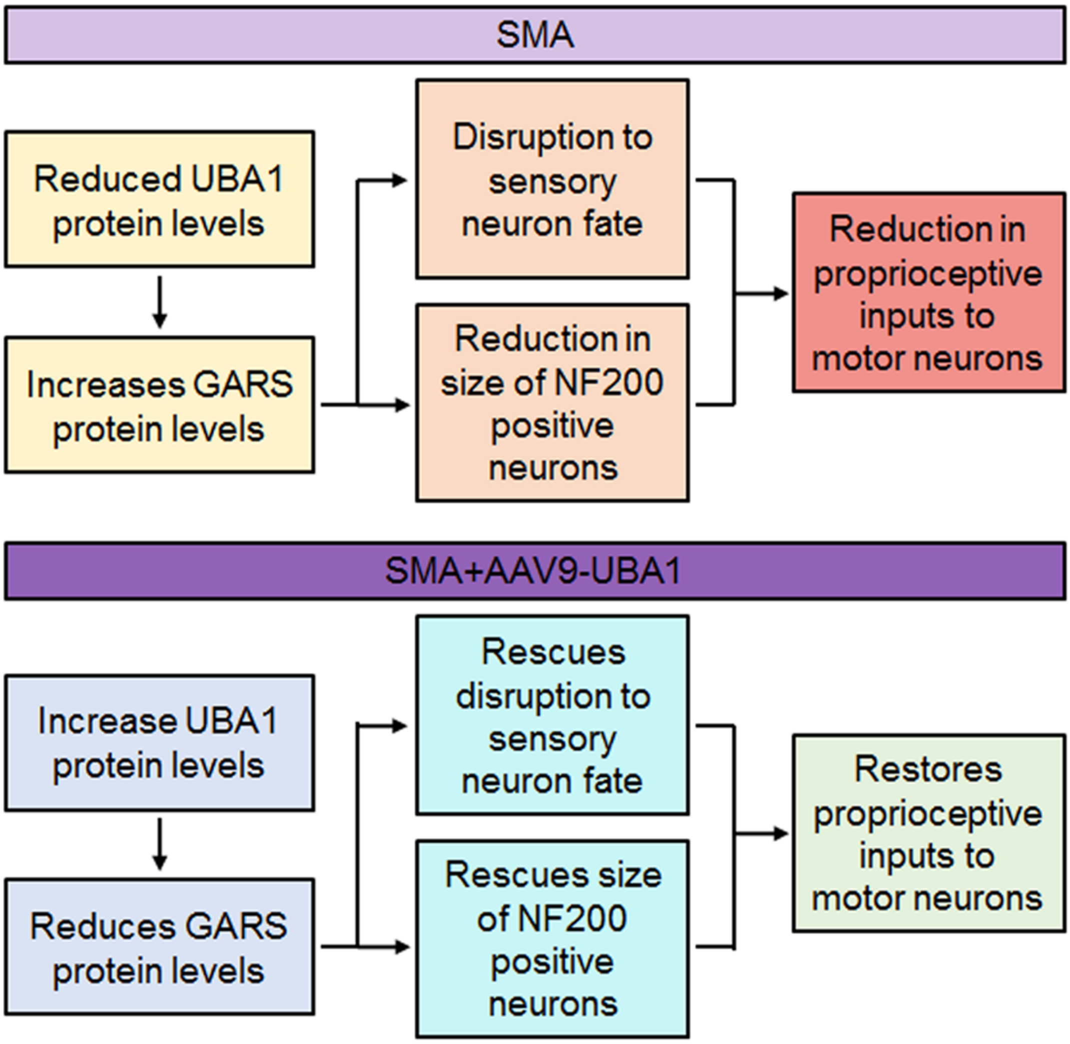

Figure S12

Disruption to sensory neuron fate and subsequent reduction in proprioceptive inputs to motor neurons is caused by a UBA1 and GARS dependent mechanism.

Table S2

Sensory pathology in SMA patients and molecular overlap of SMA and CMT.

Spinal Muscular Atrophy

Study design	***Sensory pathology***	***Genetic diagnosis***	***Reference***
Electroneuromyographic studies in 15 cases	Abnormal sensory conduction in 26.7% of patients	SMA type 1	Duman et al., 2013
Sural nerve biopsies and sensory nerve	7/7 type 1 SMA patients showed axonal degeneration	SMA type 1	Rudnik-Schoneborn et al.,
conduction studies	5/7 type 1 SMA patients showed abnormal sensory conduction	SMA type 1	2003
	0/7 type 2 and 0/5 type 3 SMA patients showed signs of sensory involvement	SMA type 2/3	
Case report - nerve conduction study	Absence of sensory responses	SMA type 1	Reid et al., 2016
Case report of 7 cases - sural nerve biopsies	7/7 showed Wallerian degeneration of biopsied sural nerves	Presumed SMA (Werdnig-Hoffmann)	Carpenter et al., 1978
	Nodules and chromatolysis in dorsal root ganglia	Presumed SMA (Werdnig-Hoffmann)	
Post mortem analysis of 9 cases	6/9 showed sensory abnormalities, 3/6 showed nodules in dorsal root ganglia	Presumed SMA	Marshall and Duchen, 1975
Case report of 2 cases	Degenerating axons in sural nerve, ballooned neurons in dorsal root ganglia	Presumed SMA (Werdnig-Hoffmann)	Murayama et al., 1991
Case report of XL-SMA patient	Nodules within dorsal root ganglia indicating sensory neuron loss	XL-SMA (*UBA1*)	Dlamini et al., 2013

Co-segregation of SMA and CMT

Study design	***Pathology***	***Genetic diagnosis***	***Reference***
Case report of 11-month-old patient	Muscle weakness and absence of sensory nerve conduction	SMA type 2 and CMT1A	Fernandez et al., 2016
Case report of patient with muscle	ENG showed signs of demyelinating neuropathy	SMA type 3 and CMT1A	Jedrzejowska et al., 2008
weakness and foot deformity	Sural nerve biopsies showed demyelinated axons	SMA type 3 and CMT1A	

Mutations that can cause SMA/SMARD1 or CMT

Disease	***Mutation***	***Pathology***	***Reference***
CMT2D	*GARS*	Peripheral neuropathy more pronounced in upper extremities including mild sensory loss	Antonellis et al., 2003
dSMA-V	*GARS*	Pure motor neuropathy affecting distal muscle groups - preferentially affecting upper extremities	Sivakumar et al., 2005
SMA	*GARS*	Infantile SMA - reduced muscle tone, weakness and muscle atrophy, no sensory involvement	James et al., 2006
CMT2Z	*MORC2*	Cramps, distal weakness, sensory impairment - sural nerve biopsies showed depletion of myelinated fibres	Sevilla et al., 2016
SMA	*MORC2*	Proximal muscle weakness, muscular hypotonia, cerebellar atrophy, normal sensory nerve action potentials	Schottmann et al., 2016
CMT2S	*IGHMP2*	Slowly progressive weakness, muscle wasting and sensory loss, no significant respiratory compromise	Cottenie et al., 2014
SMARD1	*IGHMP2*	Progressive loss of α-motor neurons, muscle atrophy and weakness, life-threatening respiratory distress	Grohmann et al., 2001
